# The ER stress response mediator ERO1 triggers cancer metastasis by favoring the angiogenic switch in hypoxic conditions

**DOI:** 10.1038/s41388-021-01659-y

**Published:** 2021-02-02

**Authors:** Ersilia Varone, Alessandra Decio, Alexander Chernorudskiy, Lucia Minoli, Laura Brunelli, Federica Ioli, Arianna Piotti, Roberta Pastorelli, Maddalena Fratelli, Marco Gobbi, Raffaella Giavazzi, Ester Zito

**Affiliations:** 1 Istituto di Ricerche Farmacologiche Mario Negri IRCCS, Milan, Italy; 2 Mouse and Animal Pathology Lab, Department of Veterinary Medicine, University of Milan, Milan, Italy

**Keywords:** Breast cancer, Metastasis

## Abstract

Solid tumors are often characterized by a hypoxic microenvironment which contributes, through the hypoxia-inducible factor HIF-1, to the invasion-metastasis cascade. Endoplasmic reticulum (ER) stress also leads tumor cells to thrive and spread by inducing a transcriptional and translational program, the Unfolded Protein Response (UPR), aimed at restoring ER homeostasis. We studied ERO1 alpha (henceforth ERO1), a protein disulfide oxidase with the tumor-relevant characteristic of being positively regulated by both ER stress and hypoxia. Analysis of the redox secretome indicated that pro-angiogenic HIF-1 targets, were blunted in ERO1-devoid breast cancer cells under hypoxic conditions. ERO1 deficiency reduced tumor cell migration and lung metastases by impinging on tumor angiogenesis, negatively regulating the upstream ATF4/CHOP branch of the UPR and selectively impeding oxidative folding of angiogenic factors, among which VEGF-A. Thus, ERO1 deficiency acted synergistically with the otherwise feeble curative effects of anti-angiogenic therapy in aggressive breast cancer murine models and it might be exploited to treat cancers with pathological HIF-1-dependent angiogenesis. Furthermore, ERO1 levels are higher in the more aggressive basal breast tumors and correlate inversely with the disease- and metastasis-free interval of breast cancer patients. Thus, taking advantage of our in vitro data on ERO1-regulated gene products we identified a gene set associated with ERO1 expression in basal tumors and related to UPR, hypoxia, and angiogenesis, whose levels might be investigated in patients as a hallmark of tumor aggressiveness and orient those with lower levels toward an effective anti-angiogenic therapy.

## Introduction

In the last decade, cancer survival rates have improved thanks to early diagnosis and new anti-cancer drugs. However, although cancer metastasis remains the cause of about 90% of cancer deaths, limited progress has been made in its treatment. Therefore, a deeper understanding of the metastasis process could offer broad potential clinical benefit by providing new targets to prevent and/or eradicate single tumor cells before metastases develop [1].

Solid tumors are often characterized by a hypoxic microenvironment and hypoxia is an adverse prognostic indicator for patients suffering from different types of cancer. Tumor cells adapt to the hypoxic environment by acting on the hypoxia-inducible factor HIF-1, which becomes functional and promotes target gene transcription [2]. In breast cancer patients, high levels of HIF-1 alpha correlate with a worse prognosis and in orthotopic transplants of human breast cancer cells in mice HIF-1alpha triggers metastasis to the lung, suggesting the high potential of hypoxic cancer cells to develop a metastatic phenotype [3].

Tumor growth and metastases also depend on angiogenesis, which supplies nutrients and leads hematogenous spread of the primary tumor [4]. Angiogenesis is stimulated by hypoxia-regulated growth factors and cytokines, including vascular growth factor (VEGF-A) which, by binding in a disulfide-bonded dimeric form to its receptor VEGFR2, triggers complex signaling, culminating in endothelial cell migration/proliferation and de novo vessel formation [5, 6]. Thus, high levels of VEGF positively correlate with the numbers of vessels in tumor sections, which are linked to the aggressiveness of breast tumors, and are also considered a negative prognostic factor for survival for this tumor type [7–9].

In addition, hypoxia disrupts endoplasmic reticulum (ER) homeostasis and leads to ER stress with consequent activation of the pro-survival ER unfolded protein response (UPR) which, by coordinating a wide array of vital cellular processes, contributes to numerous steps along the invasion-metastasis cascade in cancer [10].

As regards angiogenesis, the activated protein kinase RNA-like endoplasmic reticulum kinase (PERK) branch of the UPR promotes phosphorylation of eukaryotic initiation factor 2 alpha, with downregulation of global protein synthesis and preferential translation of the transcription factor ATF4, which promotes the upregulation of VEGF under hypoxic conditions [11]. Silencing PERK delayed tumor growth and vascularization in an orthotopic squamous carcinoma model [12], suggesting this UPR branch has an important role in regulating angiogenesis [12, 13].

Downstream of the PERK pathway of the UPR, the transcription factor CHOP promotes transcription of the gene product of the protein disulfide oxidase endoplasmic oxidoreductin 1 alpha, ERO1α (henceforth ERO1) [14]. ERO1 is strongly upregulated by hypoxic conditions [14, 15] and its high expression levels are associated with impairment of the overall survival of triple negative breast cancer patients (TNBC) [16]. This suggested investigating whether the hypoxia-mediated induction of ERO1 has significance for metastatic dissemination in patients with aggressive breast tumors.

Here, we report that the ablation of ERO1 in highly metastatic breast cancer does not massively affect the oxidative protein folding but selectively impairs that of angiogenic-related factors in hypoxic conditions, blunting metastasis. These results suggest that targeting ERO1 or the combined targeting of multiple effectors of angiogenesis might efficiently inhibit cancer angiogenesis, hence also metastases.

## Results

### ERO1 expression is higher in basal breast tumor cells

ERO1 RNA levels obtained from Cancer cell line Encyclopedia datasets indicated that ERO1 was highly expressed in several breast cancer cells (Supplementary Fig. 1A), but its level of expression was higher in basal breast cancer cells, which mostly consist of aggressive TNBC. We analyzed the protein expression of ERO1 experimentally in some of these cell lines and found that its levels were in good agreement with ERO1 RNA levels. We also found that ERO1 levels were upregulated in all cells exposed to hypoxic conditions, except for the luminal CAMA1 (Supplementary Fig. 1B). These results suggest a positive association between high levels of ERO1 and aggressive breast tumors and that hypoxia raises ERO1 levels in breast cancer cells.

### ERO1 ablation impairs migration in metastatic MDA-MB-231 cells

To study how ERO1 is involved in tumor growth and malignancy we generated human breast cancer luciferase-expressing MDAMB231 cells with enhanced tumor growth and metastases, which we referred to as MDAMB231^m^ (Supplementary Fig. 2A). In parallel, we examined a luciferase-expressing 4T1 cell line, a murine breast cancer cell line which, when orthotopically inoculated, gives a high rate of lung metastasis. Next, we generated ERO1 knock-out (ERO1 KO) in highly metastatic MDAMB231^m^ and murine 4T1 breast cancer cells using CRISPR/CAS9 technology.

**Fig. 1:**
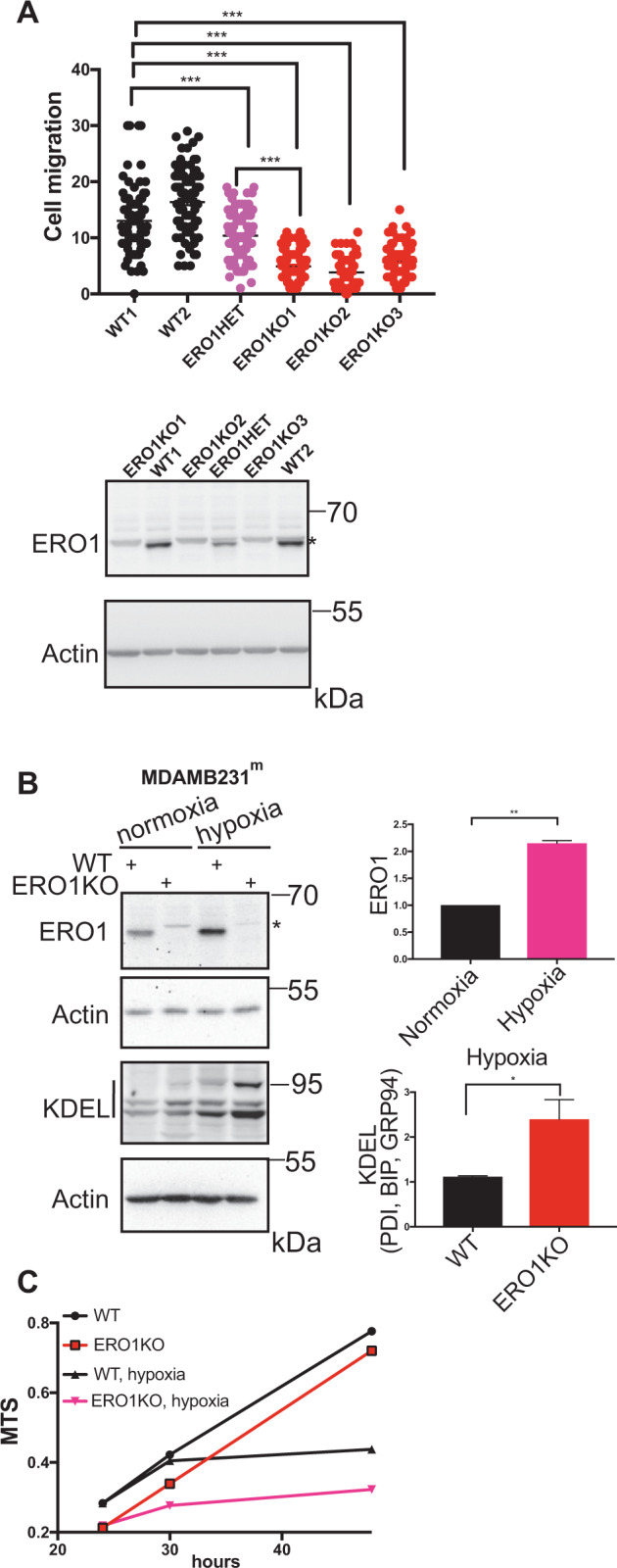
Deletion of ERO1 impairs cell migration. **A** Dot plots indicating the numbers of cells under normoxia from different clones that migrated from ten different fields of three separate Boyden chambers. Below, ERO1 immunoblot of WT, ERO1 KO and ERO1 heterozygous (HET) MDAMB231^m^ clones. Actin was used as loading control. *indicates a background band. **B** Representative ERO1 and KDEL immunoblots of WT and ERO1 KO MDAMB231^m^ exposed to hypoxia for 24 h. Actin was used as loading control. *indicates a background band. On the left, bar graphs presenting relative quantitation of ERO1 and KDEL-containing proteins (PDI, BIP, GRP94) signals. **C** Growth curve (MTS) of equal numbers of WT and ERO1 KO cells exposed to normoxia or hypoxia for 48 h (*N* = 4).

We obtained different ERO1 KO clones in both cell lines (Fig. 1A, Supplementary Fig. 3A) and analyzed them in vitro with a Boyden chamber assay for migration ability, and an MTS assay for growth rate. The lack of ERO1 impeded cell migration in a dose-dependent manner as the potential of ERO1 heterozygous cells to migrate was intermediate between that of the WT and the ERO1 KO cells (Fig. 1A and Supplementary Fig. 3B). On the other hand, the lack of ERO1, which was upregulated in hypoxic conditions, didn’t significantly impair the kinetics of MDAMB231^m^ growth either in hypoxia or normoxia (Figs. 1B, 1C Supplementary Fig. 3C).

### ERO1 ablation impairs the secretion of HIF-1 targets and angiogenesis-related factors in aggressive breast tumors

Since ERO1 is a protein disulfide oxidase [15], to assess the impact of ERO1 KO on the rate of oxidative protein folding of cells exposed to hypoxia, we analyzed the profiles of intracellular disulfide-bonded and secreted proteins by label-free proteomics of WT and ERO1 KO MDAMB231^m^ cells after exposure to normoxic and hypoxic conditions.

Proteins belonging to the protein folding network were substantially upregulated in ERO1 KO under hypoxic conditions (Supplementary Table 1), as confirmed by the immunoblot results of KDEL-containing proteins (Fig. 1B), suggesting a compensatory effect on ERO1 deficiency of these proteins involved in the folding [17].

**Fig. 2:**
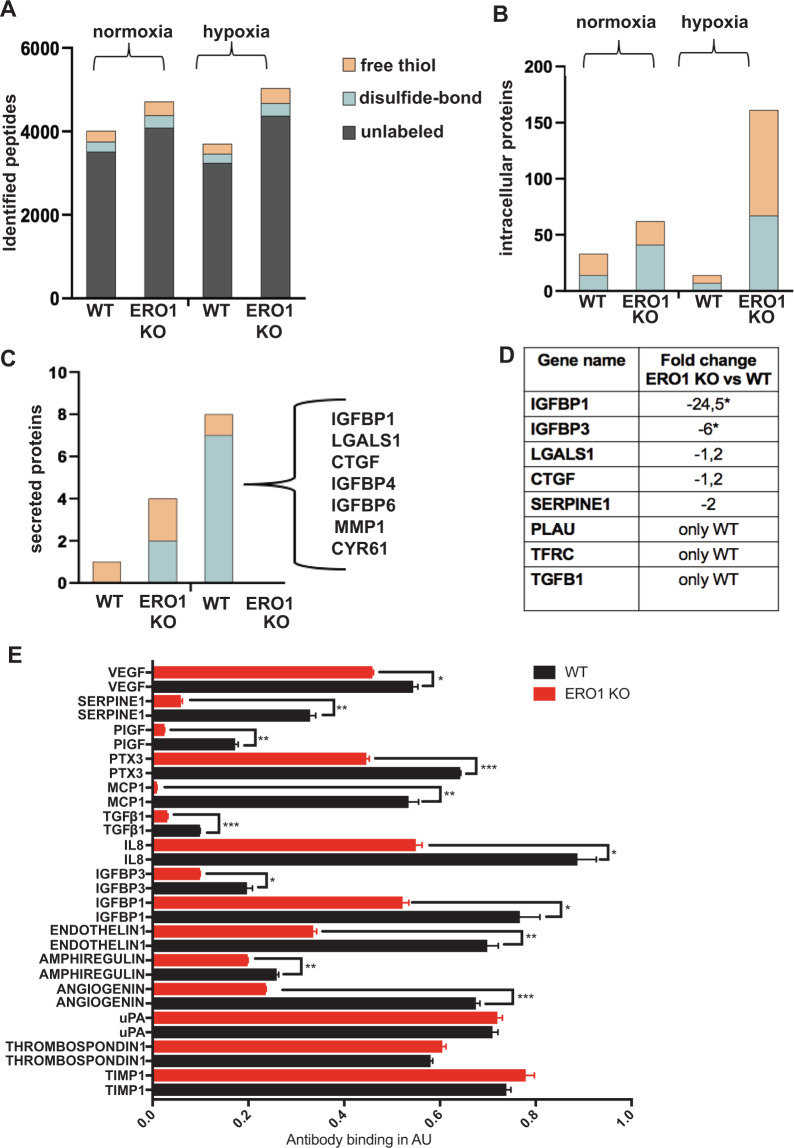
ERO1 loss impairs the secretion of HIF-1-dependent and angiogenesis-relevant targets. **A** Distribution of total intracellular peptides of WT and ERO1 KO MDAMB231^m^ under normoxic and hypoxic conditions, using nLC-MS/MS proteomic analyses. The redox state of cysteines is reported as free thiol if the cysteines are alkylated by N-ethylmaleimide (NEM) and present in a reduced form; or as disulfide-bond if the cysteines are alkylated by carbamidomethylation (IAA) and are present in an oxidized form. **B** Distribution of intracellular proteins containing differently IAA/NEM-labeled peptides in WT and ERO1 KO MDAMB231^m^. **C** Distribution of secreted proteins (secretome by nLC-MS/MS proteomic analysis) containing differently IAA/NEM-labeled peptides in WT and ERO1 KO MDAMB231^m^. Proteins containing the differently IAA-labeled peptides and identified in WT are shown as the gene name. **D** Table reporting the fold change of HIF-1 target secreted proteins as gene name (MetaCore analytical suite version 19.4) in WT and ERO1 KO MDAMB231^m^ in hypoxic conditions by secretome proteomics (nLC-MS/MS proteomic analyses) (*N* = 3). “Only WT” refers to protein identified only in the WT group. * highlighted secreted proteins whose fold change was significantly different between ERO1 KO vs. WT cells (Wilcoxon Mann–Whitney test, *p* < 0.05, JMP pro13). **E** Bar graphs presenting levels of angiogenic cytokines detected by a human angiogenic array in the secretome of WT and ERO1 KO MDAMB231^m^ undergoing hypoxia for 48 h (*N* = 3). The reference spot was set to 1. The cytokines with a value of WT above 0,1 are shown.

Nonetheless, despite quantitatively similar recovery of proteins in WT and ERO1 KO cells under hypoxic conditions (Fig. 2A), ERO1 KO cells presented more proteins containing free-thiols than WT (Fig. 2B), indicating a selective effect of ERO1 on the oxidative folding of a restricted pool of proteins.

Since secreted cytokines and growth factors stimulate endothelial cell proliferation and are associated with greater tumor aggressiveness [18], we analyzed the secretome of ERO1 KO MDAMB231^m^ in hypoxic and normoxic conditions, compared to that of the WT counterpart. In hypoxic conditions, secreted factors such as IGFBP1, LGALS1, CTGF, IGFBP4, IGFBP6, MMP1 and CYR61, which were present in a disulfide-bonded form in WT, were missing in this redox form in ERO1 KO MDAMB231^m^ (Fig. 2C), suggesting that these factors are targets of the activity of ERO1 as protein disulfide oxidase. Moreover, some of these factors, together with others such as IGFBP3, SERPINE1, PLAU, TFRC, and TGFB1 were also underrepresented in the secretome of ERO1 KO MDAMB231^m^ (Fig. 2D). This indicates a deficiency in the disulfide-bonded form and in the total amount of these soluble factors in the secretome of ERO1 KO MDAMB231^m^. Given that these are angiogenesis-related factors and are HIF-1 targets (Supplementary Table 2) [19], our results point to a role of ERO1 on a subset of secreted proteins which are angiogenesis-related.

Further analysis of the secretome of WT and ERO1 KO MDAMB231^m^ under hypoxia, by a human angiogenesis array (Fig. 2E), confirmed an impairment in angiogenesis-related cytokines in ERO1 KO MDAMB231^m^.

### ERO1 ablation impairs VEGFA in aggressive breast tumor

**Fig. 3:**
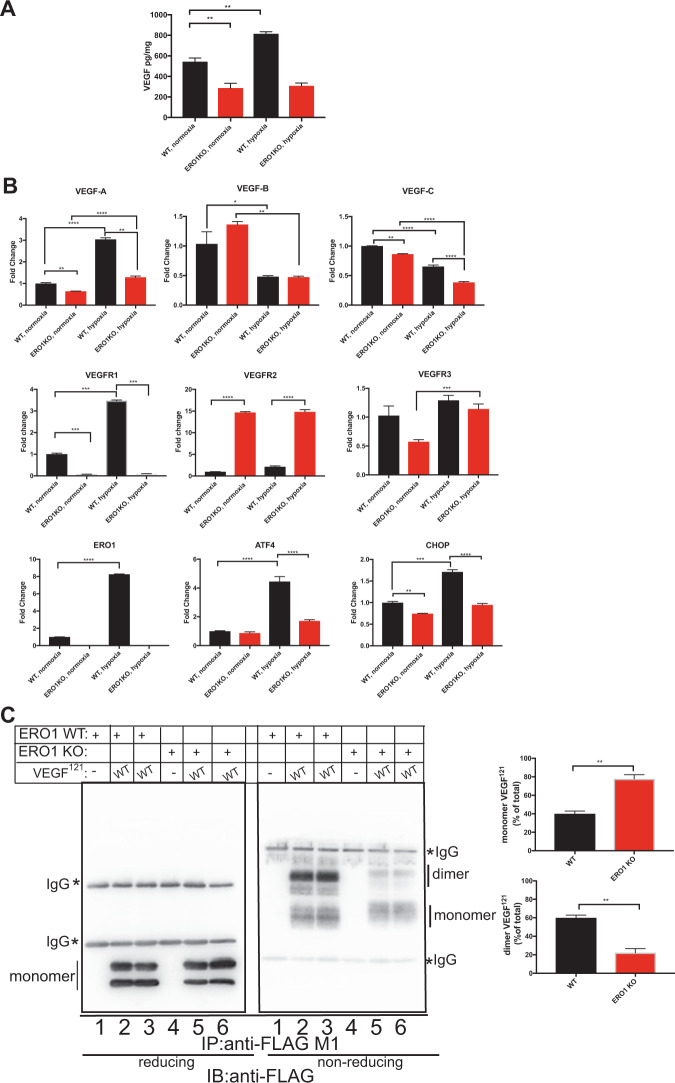

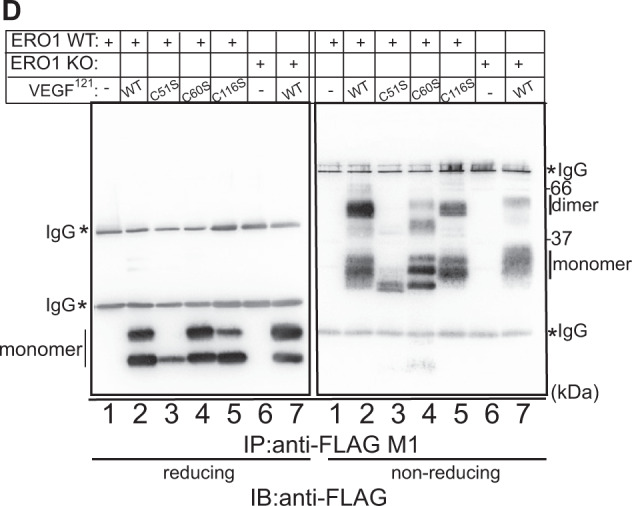
Downregulation and impaired dimerization of VEGFA in ERO1 KO tumor cells. **A** Bar graph indicating the levels of VEGF secreted by WT and ERO1 KO MDA MB231^m^ that were exposed to normoxic and hypoxic conditions (*N* = 3). **B** Bar graphs of real-time RT-PCR analysis of mRNAs (*N* = 3). **C** Reducing and non-reducing FLAG Immunoblot of FLAG-tagged VEGF^121^ (WT) immunopurified with FLAG-M1 antibody from conditioned media of WT and ERO1 KO HeLa cells that were mock-transfected or transfected with expression plasmids containing FLAG-tagged VEGFA^121^. On the right, two bar graphs indicate the levels (%) of VEGFA^121^ monomer and dimer in WT and ERO1 KO HeLa cells (*N* = 3). **D** Reducing and non-reducing FLAG-Immunoblot of the indicated FLAG-tagged VEGF^121^ (WT) and its cysteine mutants (C51S, C60S and C116S) immunopurified with FLAG-M1 antibody from conditioned media from WT and ERO1 KO HeLa cells. The monomer and dimer of VEGF^121^ are indicated. An asterisk indicates immunoglobulins (IgG).

To determine the effect of ERO1 on angiogenesis, we measured the levels of secreted VEGFA, as a paradigmatic example of an HIF-1-dependent disulfide-bonded secreted protein, in the conditioned media of WT and MDA MB231^m^ exposed to normoxic and hypoxic conditions. Levels of secreted VEGFA in conditioned medium (CM) were impaired in ERO1 KO MDAMB231^m^ compared to the WT counterpart in normoxia and the increase in VEGFA levels of WT was suppressed in the ERO1 KO MDAMB231^m^ under hypoxia (Fig. 3A); this suggests that ERO1 directly regulates VEGFA levels of MDAMB231^m^ in hypoxia. Consistently with this, quantitative analysis of the RNA levels of VEGF indicated a selective reduction of VEGFA (the isoform mainly involved in tumor angiogenesis [20]) but not of VEGFB and VEGFC, and upregulation of its receptor VEGFR2 (which might indicate a compensatory mechanism following reduced VEGFA) in ERO1 KO MDAMB231^m^ under hypoxia (Fig. 3B).

ATF4 and CHOP, which belong to the PERK pathway of the UPR/ER stress response [21], and are upstream to ERO1 regulation [14], were both downregulated in ERO1 KO cells under hypoxia (Fig. 3B). Further analysis showed steady increases in ATF4 and CHOP in WT cells during hypoxia that was not observed in ERO1 KO cells (Supplementary Fig. 2B). This might be due to a feedback loop between ERO1 levels and the levels of the upstream CHOP and ATF4, which are involved in the transcription of VEGF, the angiogenic switch and cancer metastases [22].

### Impaired dimer-to-monomer ratio of VEGFA in ERO1-devoid breast tumors

ERO1 is a protein disulfide oxidase of the ER that together with PDI introduces disulfide bonds in new client proteins, and VEGFA is secreted by cells as a disulfide-bonded homodimer, which is the isoform able to interact with its receptor. We, therefore, tested whether a lack of ERO1 affected the disulfide-bonded VEGF homodimers [15, 23–25], though not detected among the secreted disulfide-bonded proteins of Fig. 2C.

To study the biogenesis of VEGF in WT and ERO1 KO HeLa and MDAMB231^m^ cells, we engineered a FLAG-expression vector encoding VEGF^121^, a soluble splicing variant of VEGFA involved in angiogenesis. We tested the intracellular and secreted contents of this VEGF isoform under reducing and non-reducing immunoblot, which might point to differences in the homodimeric state of VEGFA in ERO1 KO cells [26]. VEGF^121^ was barely detectable intracellularly, suggesting its fast secretion (data not shown) [16]. However, it was secreted (as presumably two differently glycosylated isoforms) equally by WT and ERO1 KO cells (lanes 2–3 vs. lanes 5–6 from the immunoblot in reducing conditions of Fig. 3C and Supplementary Fig. 2C). In the non-reducing immunoblot FLAG-VEGF^121^ secreted from WT cells appeared in two bands, corresponding to a faster-migrating form (apparent Mw below 37 kDa), presumably a glycosylated VEGF monomer, and a predominant form (apparent Mw around 66 kDa), compatible with a glycosylated homodimer (Fig. 3C and Supplementary Fig. 2C). However, the slow-migrating VEGF homodimer that is disulfide-bonded, as it was not visible in reducing conditions, was barely detected in ERO1 KO cells (Fig. 3C, lanes 2–3 vs. lanes 5–6 in non-reducing conditions). This suggests that the lack of ERO1 promotes the secretion of mostly VEGF monomer (80% of the total in HeLa cells).

To investigate the effect of ERO1 on VEGF homodimerization, we created a panel of FLAG-tagged VEGF^121^ mutants in the cysteines 51, 60 and 116 (C51S, C60S and C116S) that are involved in VEGF homodimerization [23]; we then expressed them in WT HeLa cells and analyzed these mutants under reducing and non-reducing immunoblot (Fig. 3D). VEGF^121^C51S was unstable as in the reducing condition it was recovered at a low level (lane 3, Fig. 3D). VEGF^121^C60S and C116S had less ability to homodimerize, with patterns similar to the parental VEGF^121^ in ERO1 KO cells (compare lanes 2, 4, 5 with lane 7 in non-reducing conditions, Fig. 3D). This indicates that the lack of ERO1 selectively impaired the secretion of VEGF^121^ homodimer, which is mediated by cysteines 60 and 116 (Fig. 3D).

**Fig. 4:**
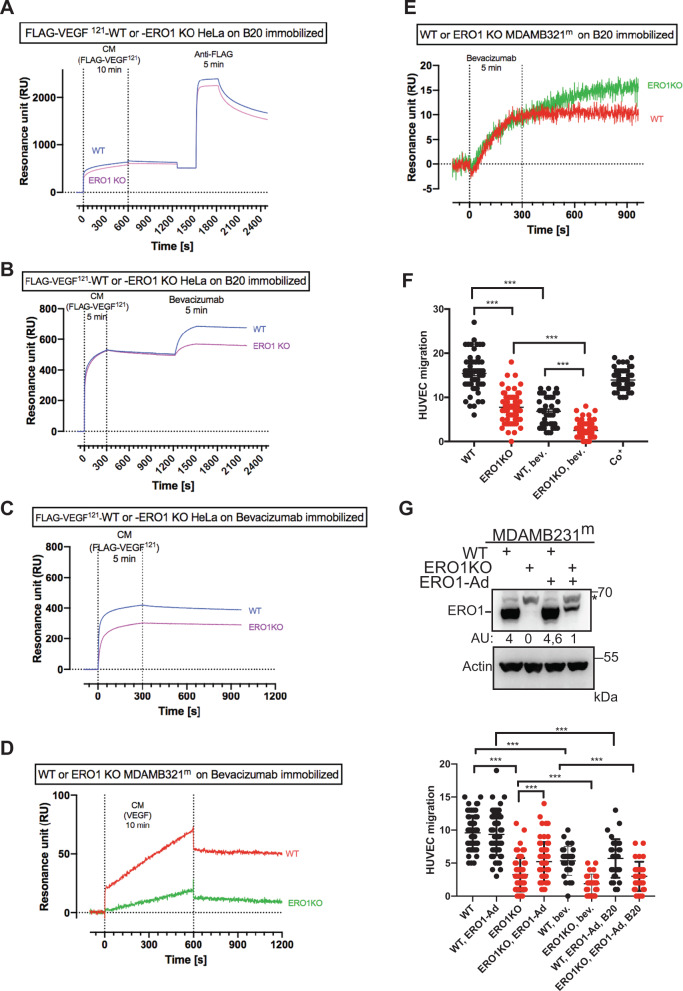
ERO1 loss affects endothelial migration by impairing VEGF homodimers. **A–E** Sensorgrams (time course of SPR signals in Resonance units, RU) of VEGF signal obtained by flowing CM for 5–10 min (as indicated) on immobilized anti-VEGF antibody B20 (**A**, **B**, **E**) or bevacizumab (**C**, **D**). The injection of CM from FLAG-VEGF ^121^-WT and -ERO1 KO HeLa cells over immobilized B20 was followed by the injection of 10 µg/mL of anti-FLAG (**A**) or Bevacizumab (**B**). Injection of bevacizumab over B20-captured VEGF from CM of WT- and ERO1 KO -MDAMB231^m^ cells (under hypoxic conditions) (**E**). **F** Dot plots indicating the number of HUVEC migrated toward the conditioned media from WT and ERO1KO MDAMB231^m^. Co^+^ is a positive control, i.e., conditioned media from NIH3T3 fibroblasts that was used as an attractant for HUVEC. **G** Immunoblot of ERO1 in WT and ERO1KO MDAMB231^m^ cells after infection with an ERO1-adenovirus (ERO1-Ad.) or an inert GFP-adenovirus. * indicates a background band. Quantitation of the signal is indicated in an arbitrary unit (AU). Below, dot plots indicating the number of HUVEC cells that migrated toward the conditioned media from equal numbers of WT and ERO1KO MDA MB231^m^ infected with an ERO1-adenovirus (ERO1-Ad.) or an inert GFP-adenovirus. Bev. and B20 indicate Bevacizumab and B20 respectively, two neutralizing antibodies against VEGF.

We subsequently exploited surface plasmon resonance (SPR) to determine the relative abundance of monomers and dimers of VEGF(A) in CM from WT and ERO1 KO MDAMB231^m^ under hypoxia. To this end, these two VEGF conformers were discriminated by their binding to two neutralizing antibodies of VEGF, B20 and bevacizumab, immobilized on the sensor chip. The assay was initially calibrated on FLAG-VEGF^121^ from CM of WT and ERO1 KO HeLa cells, that is secreted in a comparable amount between the two different cells but present prevalently in a dimeric form in WT and in a monomeric form in ERO1 KO cells (Fig. 3C). The CM of these cells resulted in a similar VEGF-specific SPR signal on immobilized B20 (Fig. 4A). Consistently, the following injection of FLAG antibody resulted in a similar SPR signal between the secreted VEGFs from the two different cells (Fig. 4A). This confirms a comparable amount of total VEGF in the CM of WT and ERO1 KO HeLa cells and suggests that B20 recognizes monomers and dimers with a comparable affinity. However, when Bevacizumab was injected after the CM a higher binding signal was observed in the CM of WT cells (Fig. 4B) suggesting a preferential binding of Bevacizumab for the VEGF dimers. This was further confirmed by injecting CM on immobilized bevacizumab, that also points to a higher SPR signal of CM from WT cells (Fig. 4C).

In CM of MDAMB231^m^ under hypoxic conditions, the VEGF-dependent binding on immobilized bevacizumab was higher for WT (Fig. 4D), whereas VEGF in CM of WT and ERO1 KO MDAMB231^m^ showed a comparable binding on immobilized B20 (Fig. 4E). These results indicate a larger proportion of dimers to monomers VEGF (captured by Bevacizumab) in CM of WT than ERO1 KO and a comparable amount of total VEGF epitope (captured by B20) in CM of WT and ERO1 KO MDAMB231^m^.

### ERO1 loss negatively affects angiogenesis

To investigate the ability of VEGF secreted by ERO1 KO cells to induce migration of endothelial cells we employed CM collected from equal numbers of WT and ERO1 KO MDAMB231^m^ to induce migration of HUVECs, which are primary endothelial cells with a proangiogenic potential (as they express pro-angiogenic factors such as the VEGFR2 receptors). We analyzed CM from WT and ERO1 KO MDAMB231^m^ with or without bevacizumab and B20 for their ability to promote HUVEC migration, a fundamental step of angiogenesis. In good accordance with a more active (dimeric) VEGF secreted by WT cells, CM from WT cells stimulated HUVEC migration to a greater extent than CM from ERO1 KO cells. Furthermore, inhibition of VEGF in the CM pretreated with the VEGF-neutralizing antibodies B20 and bevacizumab reduced HUVEC migration to a similar extent as the CM from ERO1 KO cells (Fig. 4F, G). Then, reintroducing ERO1 through an adenoviral infection (ERO1-Ad) in MDA MB231^m^, from which the CM had been collected, rescued the HUVEC migration defect (Fig. 4G). This suggests that the angiogenic potential of ERO1 KO CM is impaired because the low content of active VEGF.

### ERO1 regulates metastasis formation in malignant breast tumors

To study the consequence of the lack of ERO1 in the angiogenesis and metastatic spread of breast cancer, we injected luciferase-expressing human WT and ERO1 KO MDAMB231^m^ as well as murine WT and ERO1 KO 4T1 cells, in orthotopic sites of mice, and longitudinally followed the tumor growth and metastatic spread in the two different breast cancer models by in vivo bioluminescence imaging.

**Fig. 5:**
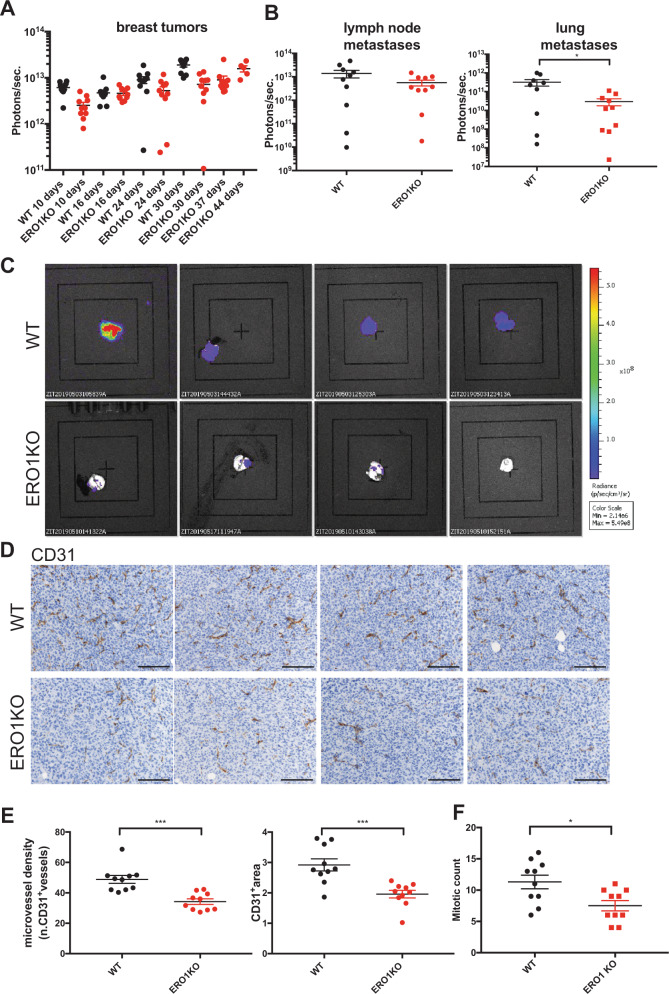
ERO1 loss impairs lung metastases by acting on angiogenesis. **A** Dot plots on a logarithmic scale of the bioluminescence counts of primary breast tumor growth, **B** lymph node and lung metastases three weeks after surgical removal of equal-sized WT and ERO1 KO MDAMB231^m^ primary breast tumor in orthotopically injected mice (each dot indicates one mouse) (*N* = 10). **C** Bioluminescence signals of lungs from four representative mice. **D** Representative micrographs of CD31 IHC staining in primary tumors (scale bar 100 µm), **E** relative quantification of CD31^+^ blood vessels and (**F**) mitotic counts (*N* = 10).

ERO1 KO breast tumors grew very similarly to WT when in the 4T1 background but slightly more slowly than the WT counterpart when in MDA MB231^m^ (Supplementary Fig. 3D and Fig. 5A).

To assess the metastatic potential of ERO1 KO and WT tumors, the primary tumors were surgically removed when they reached similar size and metastases were analyzed. Mice bearing ERO1 KO tumors had between 10 and 50 times fewer lung metastases than the WT counterparts (Supplementary Fig. 3E and Fig. 5B-C), suggesting a main effect of ERO1 on the metastatic spread. Interestingly, there was no difference in artificial metastasis dissemination between tumor-bearing mice tail vein-injected with WT or ERO1 KO 4T1 cells (Supplementary Fig. 3F); this implies that the reduced metastatic dissemination depends on the lack of ERO1 in the primary tumors.

We also analyzed the role of ERO1 in the stroma, which represents the tumor microenvironment. E0771 breast cancer cells were transplanted orthotopically in the mammary fat pad of syngeneic WT and ERO1 KO mice and the tumor development and progression between them compared over time. Growth curves suggested that primary tumors grew slightly faster in WT than ERO1 KO mice (Supplementary Fig. 3G). Furthermore, although the E0771 cells have low metastatic potential, there were more lung metastases in WT than ERO1 KO mice (Supplementary Fig. 3H). Thus the lack of ERO1 in both the primary tumor and the stroma impairs tumor growth and dissemination.

### ERO1 loss synergizes with anti-angiogenic treatment in delaying tumor progression and metastases

To correlate the ERO1 KO-mediated impairment in metastatic spread of MDA-MB231^m^ with defective angiogenesis, we immunostained the primary tumors with CD31, an endothelial marker (Fig. 5D). Quantification of the stain indicated a 30% reduction in microvessel density (Fig. 5E) and a 40% lower tumor mitotic count (Fig. 5F) in ERO1 KO tumors, suggesting some impairment in the tumor vasculature of ERO1-devoid breast tumors. These data suggest that ERO1 loss in breast tumors might inhibit lung metastasis spread by impairing angiogenesis.

**Fig. 6:**
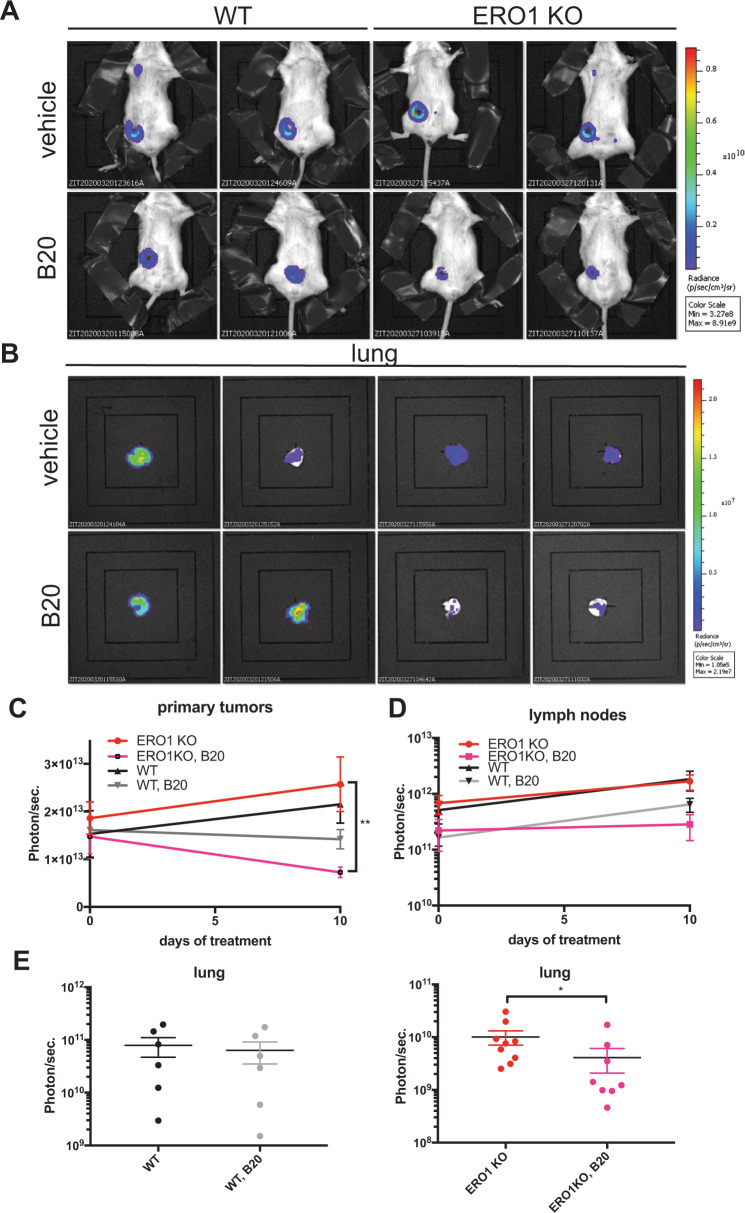
Anti-angiogenic therapy is effective in ERO1-devoid breast tumors. **A** Bioluminescence signals of primary breast tumors and lungs from representative mice orthotopically-injected with WT and ERO1 KO MDA-MB231^m^, and treated three times with the anti-VEGF, B20 or vehicle. **B** Bioluminescence signals of ex vivo lungs. **C** Primary breast tER stress response mediator induced by hypoxia. Its lack impairsumor and (**D**) lymph node growth. **E** Dot plots on a logarithmic scale of the bioluminescence counts of lungs after B20 or vehicle treatment.

Given the influence of ERO1 on angiogenesis, we analyzed the effect of anti-angiogenic therapy with the VEGF neutralizing antibody B20, which accordingly to our SPR results, binds VEGFA monomers and dimers comparably, on breast tumors of equal size of mice injected with WT and ERO1 KO MDAMB231^m^ in orthotopic sites. B20-treated ERO1 KO MDAMB231^m^ injected mice had a significant—50% decrease- in breast tumor growth compared to the vehicle-treated counterparts; in contrast, as expected, B20-treated WT MDAMB231^m^-injected mice had a scant, not statistically significant average decrease (18%) in breast tumor growth compared to the vehicle-treated counterpart (Figs. 6A and 6C). Furthermore, despite the non-significant difference in the lymph node metastasis burden in vehicle- and B20-treated ERO1 KO MDAMB231^m^ mice (Fig. 6D), there were three times fewer lung metastases in B20-treated ERO1 KO MDAMB231^m^ mice, but no effect on lung metastasis in the WT counterpart (Figs. 6B and 6E). This indicates that anti-angiogenic therapy is more effective in delaying tumor growth and reducing lung metastatic burden in ERO1 KO MDA-MB231^m^-injected mice, thus suggesting its therapeutic potentiation on ERO1-impaired tumors.

### Expression of ERO1 is a biomarker for aggressive breast tumors

**Fig. 7:**
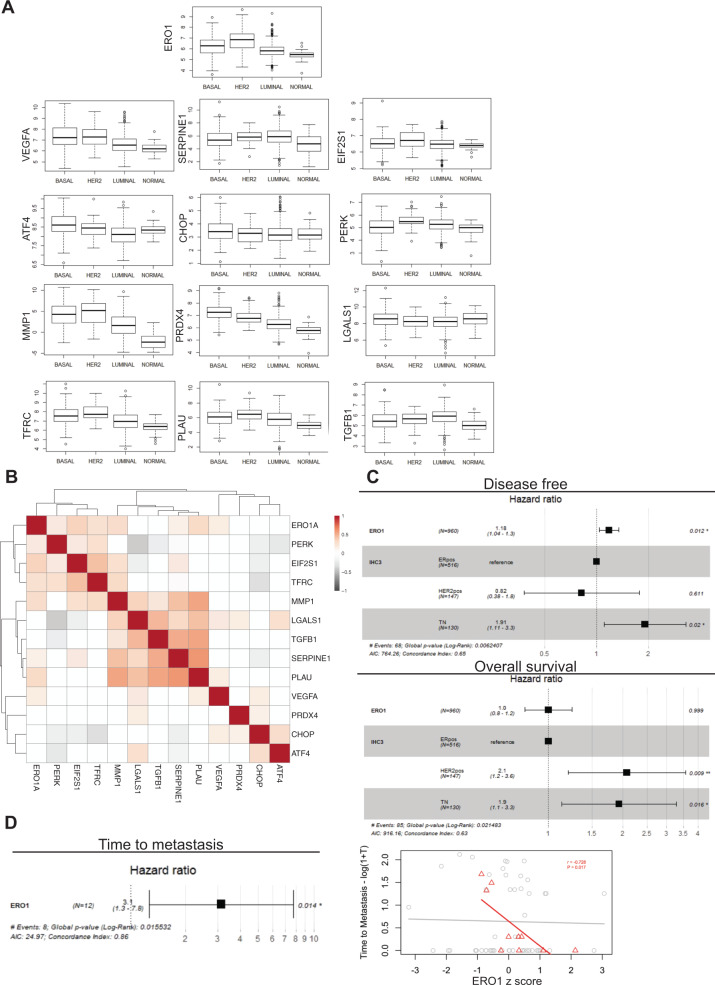
ERO1-related gene network in breast tumors as a biomarker for tumor outcome. **A** Box plots of the mRNA expression profiles of the indicated genes from 1098 cases of human breast cancer from The Cancer Genome Atlas (TCGA) [27], classified in the three subtypes: basal, luminal and Her2^+^, according to PAM50 (PAM50 is a 50-gene signature that classifies breast cancer in different molecular intrinsic subtypes: luminal, HER2-enriched, Basal, and Normal-like), along with samples from normal breast tissue. **B** Heat map of the significant correlations (*p* < 0.05) among the levels of expression of the same genes within basal tumors. **C** Forest plots for the change in the risk for recurrence and all cause death for each unit increase in ERO1 expression after adjusting for tumor subtype (IHC: Immunohistochemistry, ERpos: Estrogen receptor positive, HER2pos: Her2 positive and TN: Triple Negative) in TCGA breast cancer patients. **D** On the left: Forest plot for the change in the risk of metastasis for each unit increase in ERO1 expression in metastatic Triple Negative breast cancers from the MBC project. On the right: Scatter plot of ERO1 levels in the primary tumor as a function of the time to metastasis in all (gray circles) and Triple Negative (red triangles) breast cancers from the same dataset. Pearson correlation (P) significance in indicated.

To study the relevance of these findings in patients we analyzed ERO1 levels in different breast cancer subtypes in humans to see whether ERO1 levels influence the outcome of the disease. We analyzed the mRNA expression profiles of 1098 cases of human breast cancer from The Cancer Genome Atlas (TCGA) [27], classified in the three subtypes: basal, luminal and Her2^+^, according to PAM50, along with samples from normal breast tissue. Exploration of the expression landscape of the three breast cancer subtypes revealed higher expression levels of ERO1 in the more aggressive basal subtype, as it was also seen in cells. Interestingly, a similar expression pattern in the different breast cancer subtypes was identified for some genes that we found co-regulated or regulated by ERO1 in our in vitro studies and belonging to the angiogenesis (VEGFA, SERPINE1, MMP1, LGALS1, TGFB1, TFRC, PLAU), to the PERK/ATF4/CHOP pathway of UPR (PERK/EIF2AK3, ATF4, CHOP/DDIT3 and EIF2 alpha/EIF2S1) and the ERO1’s cognate, PRDX4 [17]. However, an opposite pattern was identified for other of them (VEGFR2/KDR, CYR61/CCN1, IGFBP1, IGFBP4, IGFP6) and no changes for CTGF/CCN2, and IGFP6 across the breast cancer subtypes (Fig. 7A and Supplementary Fig. 4A and B).

In addition to the pattern of expression in the cancer subtypes, we explored the correlations of the expression of these genes within basal tumors. The heat map (Fig. 7B) shows the significant correlations between ERO1 and PERK, EIF2 alpha, VEGFA, SERPINE1, PLAU, TFRC and MMP1 in basal tumors, confirming the strong association of ERO1 with the PERK branch of UPR and angiogenesis in basal breast cancer (Fig. 7B).

Cox proportional hazards regression analysis of ERO1 on the disease-free interval showed that patients with higher expression of ERO1 had a higher risk of tumor recurrence after adjusting for tumor subtype (HR 1.18 for each unit increase in ERO1 levels, 95% CI 1.04–1.30, *p* = 0.012) and tumor grade (HR 1.20, 95% CI 1.03–1.40, *p* = 0.015).

The association with overall survival was less clear, as it was not significant after accounting for tumor subtype (HR 1.00, 95% CI 0.80–1.20, *p* = 0.999) and significant when adjusting for tumor grade (HR 1.20, 95% CI 1.03–1.40, *p* = 0.020) (Fig. 7C). Furthermore, analysis of samples from the Metastatic Breast Cancer project (Provisional, February 2020) indicated an inverse correlation between ERO1 levels in the primary tumor and the time from diagnosis to detection of the first metastases in aggressive Triple negative tumors (Fig. 7D) suggesting a correlation between ERO1 levels and the metastatic spread from the breast tumor.

Therefore in this study we combined a target analysis with a correlation analysis on breast tumors to identify a small set of ERO1-dependent genes that might influence the tumor outcome and could serve as biomarkers of tumor aggressiveness in patients with basal breast tumors.

## Discussion

ERO1 is the predominant protein disulfide oxidase, that together with PDI introduces disulfide bonds in new client proteins [15] and its expression is positively regulated by hypoxia and ER stress [28], two hallmarks of malignant breast cancers [29, 30]. Here, we show that ERO1 expression is higher in the more aggressive basal breast tumors than in luminal tumors or normal breast tissue suggesting a role of ERO1 in tumor aggressiveness [16, 31]. However, the activity of disulfide-bond formation of ERO1, which might contribute to the folding of proteins during tumor growth and dissemination, is compensated by other pathways also involved in disulfide-bond formation [17, 25, 32, 33], questioning a defect in oxidative protein folding and thus impairment in tumor growth and spread with the interference of ERO1 activity. Indeed, our proteomic analysis indicated substantial upregulation of the protein folding pathway in ERO1 KO MDAMB231^m^ under hypoxia, and this might serve as a compensatory cellular mechanism to carry out oxidative folding in the absence of ERO1. However, analysis of the secretome of highly aggressive MDAMB231^m^ identified a selective effect of the lack of ERO1 on the oxidative status of cysteines and on the secretion of a group of disulfide-bonded and HIF-1 targets relevant for angiogenesis; this suggests that, despite compensation, a subset of proteins is still oxidatively folded *via* ERO1.

VEGF contains intramolecular disulfide bonds in its monomeric form and the intracellular ratio of oxidized to reduced VEGF monomers was impaired in ERO1-deficient cells [16]. However, we found that the secreted receptor binding-competent and disulfide-bonded dimer of one of the VEGFA isoforms, VEGF_121_—among the most important HIF-1-dependent regulators of angiogenesis—is impaired but its monomeric form was still secreted by ERO1 KO breast cancer cells. This excludes any massive unfolding due to an intramolecular lack of disulfide bonds, of the VEGFA monomer and suggests the thought-provoking hypothesis that ERO1 function is essential only for a subset of disulfide bonds, i.e., the post-translational ones involved in VEGF dimerization.

We also identified a feedback loop between ERO1 and its upstream UPR mediator, the transcription factor ATF4, which was previously identified in the angiogenic switch of aggressive tumors by regulating VEGFA expression levels [12]. This might suggest some indirect transcriptional control of ERO1 on VEGFA and other angiogenic factors.

Although we still do not know the mechanistic basis of ERO1 selectivity for angiogenic-related targets or the feedback loop between ERO1 and the transcription factor ATF4, these findings do support the notion that in hypoxic conditions ERO1 promotes the secretion of active angiogenic factors at multiple levels, i.e., directly by promoting their oxidative folding and indirectly by regulating their levels. Accordingly, ERO1 KO breast tumor cells had lower pro-angiogenic potential, as was also seen for ERO1-deficient hepatocarcinoma cells [34], and ERO1-devoid metastatic breast tumors had few blood vessels in the primary tumors, with fewer distant lung metastases.

Reduced lung metastasis is likely to be the consequence of the defective angiogenesis of the primary tumor that prevents the access of tumor cells into the circulation and their dissemination to the lung, but also results from the reduced ability of ERO1 KO cancer cells themselves to migrate to the lung.

**Fig. 8:**
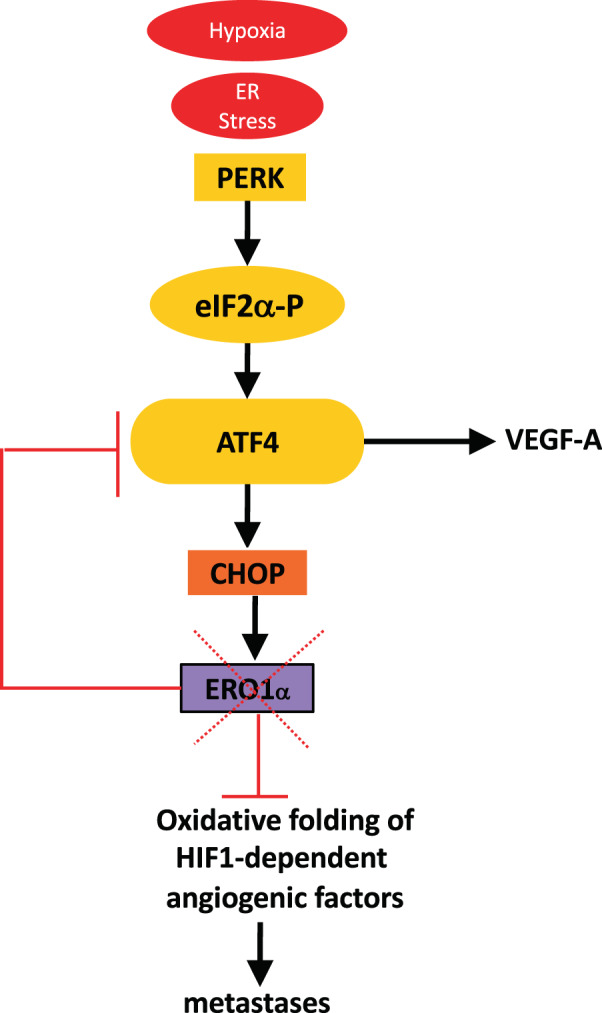
ERO1-related metastatic potential. ERO1 is an ER stress response mediator induced by hypoxia. Its lack impairs the tumor angiogenic switch by down-regulating the upstream transcription factor ATF4 and impeding the oxidative folding of angiogenesis-related factors in hypoxic conditions, such as the receptor-competent disulfide bond homodimer of VEGFA. This reduces metastatic spread and boosts the effects of anti-angiogenic therapy in breast cancer.

Furthermore, the VEGF neutralizing antibody B20 reduced tumor size and lung metastases in ERO1-devoid xenograft mouse models of aggressive breast tumors, whereas in the WT counterpart this treatment had scant effects (Fig. 8). In view of the complexity of angiogenesis, involving so many pro-angiogenic mediators, and rationalizing the failure of anti-VEGF as monotherapy in cancer [35], it is conceivable that affecting angiogenesis at several levels, such as that imposed by ERO1 deficiency, is more effective than the impairment of only one mediator for the treatment of breast tumors and their metastases.

In conclusion, the clear correlation between high ERO1 levels and the more aggressive form of basal breast tumors suggests that ERO1 analysis together with the analysis of the ERO1-related gene set belonging to the HIF-1, UPR and angiogenesis pathway, as proposed here, might be helpful to predict the outcome for basal breast cancer patients. Envisaging a future of personalized precision medicine for cancer therapy, we hypothesize that those patient tumors with low expression of these genes—hence with with an impaired angiogenic signature—might find anti-angiogenic therapy more effective.

Although at the moment the lack of a selective ERO1 inhibitor that works in vivo prevents us treating tumors and metastases [36], our findings provide a mechanistic basis for their pharmacological treatment by acting on angiogenesis at multiple levels through direct or indirect ERO1 inhibition.

## Materials and methods

### Tumor cell lines

Cells were kept in culture for no more than 2 weeks and routinely tested for mycoplasma infection. MDAMB231^m^ cells were selected from parental MDAMB231 (#505366 from ATCC Frederick Cancer Tumor Repository, Maryland, USA) through passages in mice to enhance their tumorigenic and metastatic properties as described in [37]. 410.4 cells (4T1) were provided by Amy M. Fulton (Detroit, Michigan, USA) and E0771 were bought (940001-A from CH3 BioSystems). MDAMB231, MDAMB231^m^, 4T1 and E0771 cells were infected with a lentiviral vector carrying the coding sequence of the synthetic firefly luciferase gene, luc2 (*Photinus pyralis*). Twenty-four hours after infection, cells were selected with blasticidin (5 ug/ml). Primary cultures of endothelial cells (human umbilical vein endothelial cells [HUVECs]) were isolated from umbilical cord veins [38] and grown on 1% gelatin-coated flasks in M199 supplemented with 10% FBS, 10% newborn calf serum, 20 mM Hepes, 2 mM glutamine, 6 U/mL heparin, 50 µg/ml endothelial cell growth factor, penicillin, and streptomycin. Cells were used between the third and fifth passages.

### Cell culture and transfection

Human MDAMB231^m^ and murine 4T1 breast cancer cells were transfected with ERO1-Lα CRISPR-Cas9 KO plasmids (SC-401747 for human and SC-424456 for murine, Santa Cruz Biotechnology) with three target-specific guide RNAs (gRNA). ERO1 KO HeLa cells were described in [39].

### Western blotting

Cells were lysed in cold buffer containing 150 mM NaCl, 20 mM HEPES pH 7.5, 10 mM EDTA and 1% Triton X100, supplemented with a protease inhibitors cocktail (Roche) and 20 mM NEM. Protein samples separated by either reducing or non-reducing SDS-PAGE were then transferred to Protran nitrocellulose membrane (Merck) and probed with the following antibodies: monoclonal mouse anti-Actin (MAB1501, Sigma Aldrich), monoclonal mouse anti-KDEL (ADI-SPA-827, Enzo life Sciences), monoclonal mouse anti-FLAG M2 (F3165, Sigma Aldrich), and polyclonal rabbit anti-ERO1 alpha [40].

### VEGF ELISA and angiogenesis array

Secreted VEGF and angiogenesis-related cytokines were measured in the conditioned media of MDAMB231^m^ cells by human VEGF Quantikine ELISA Kit (DVE00, R&D Systems) and human Angiogenesis Array (ARY007, R&D Systems) respectively.

### Surface plasmon resonance (SPR)

The ProteOn XPR36 Protein Interaction Array system (Bio-Rad Laboratories, Hercules, CA) was used for these studies. VEGF neutralizing antibodies B20 and Bevacizumab as well as IgG from human serum (I4506, Sigma) were covalently immobilized in three parallel channels of the same sensor chip (GLC, Bio-Rad) by amine coupling chemistry [41].

### Animals

Eight- to ten-week-old female SCID and BALB/cByJ mice were obtained from Charles River Laboratories (Calco, Italy) and maintained under specific-pathogen-free conditions. SCID mice were housed in isolated vented cages, and handled using aseptic procedures. The ERO1 alpha KO mouse line was resuscitated from the embryos of David Ron’s stock. WT and ERO1 KO C57BL/6J mouse were bred in our animal facility and genotyped according to a previously described protocol [42]. Procedures involving animals and their care were conducted in conformity with the following laws, regulations and policies governing the care and use of laboratory animals: Italian Governing Law (D.lgs 26/2014, authorization number19/2008-A issued 6 March 2008 by Ministry of Health; authorization 773–2019PR and 395/2018PR to E.Zito); Mario Negri Institutional Regulations and Policies providing internal authorization for people conducting animal experiments (Quality Management System Certificate—UNI EN ISO9001: 2008—registration number 6121); the NIH Guide for the Care and Use of Laboratory Animals (2011 edition); EU directives and guidelines (EEC Council Directive 2010/63/UE), and in line with Guidelines for the welfare and use of animals in cancer research [43].

### Breast tumor models

For the therapy experiment G*Power, version 3.1.9.2, was used to calculate the power analysis. In general, ten mice were inoculated with WT- and ERO1 KO-MDAMB231^m^ in the m.f.p., and when the tumor reached around 150–200 mm^3^ mice were randomized based on tumor weight to receive B20 or saline. B20 was diluted in saline before use and administered intravenously (i.v.) every 4 days at the dose of 5 mg/kg. Twenty-four hours after three treatments mice were sacrificed, and primary tumors and metastases quantified by BLI by an operator who was blinded to the group allocation.

### Trascriptomics

RNAseq Cancer Cell Line Encyclopedia [44] ERO1 mRNA z-score expression levels of breast cancer cell lines were retrieved from the CBioPortal for Cancer Genomics website (https://www.cbioportal.org/) [45].

### Statistics

Data are represented as mean ± SEM and were analyzed by Prism 7 (Graphpad). N was indicated in the figure legends except for dot plots. One-way ANOVA multiple comparison tests was used for analysis of Fig. 1A, the unpaired *t*-test for statistical analysis of Figs. 1B and 2E, one-way ANOVA multiple comparison tests for analysis of Figs. 3A and 3B, the unpaired *t*-test for Fig. 3C and one-way ANOVA multiple comparison tests for Figs. 4F and 4G. Samples distribution in Fig. 5B did not follow a normal distribution (Shapiro–Wilk normality test) hence the non-parametric Kolmogorov–Smirnov test was applied for the analysis. The unpaired *t*-test was used for Figs. 5E, 5F, 6C, and 6D. Samples distribution in Fig. 6E did not follow a normal distribution (Shapiro–Wilk normality test) hence the non-parametric Kolmogorov–Smirnov test was applied for the analysis. The unpaired *t*-test was used for Supplementary Fig. 1A, one-way ANOVA multiple comparison tests for Supplementary Fig. 2B and the unpaired *t*-test for Supplementary Fig. 2C, 3B-D, F-H. One asterisk indicates *p* < 0.05, two for *p* < 0.01, three for *p* < 0.001 and four for *p* < 0.0001.

## Supplementary information

Supplemental figures and Material

Supplemental table 1

Supplemental table 2

## Data Availability

The authors confirm that the data supporting the findings of this study are available within the article [and/or] its supplementary materials and are deposited in the public repository Zenodo (10.5281/zenodo.4251007).
