# Supplementary material for: The ER stress response mediator ERO1 triggers cancer metastasis by favoring the angiogenic switch in hypoxic conditions
Source: Oncogene. 2021 Feb 2;40(9):1721–36. doi: 10.1038/s41388-021-01659-y (PMC7932925; doi:10.1038/s41388-021-01659-y)
Supplement: Supplementary file 1 — Supplemental figures and Material [file 41388_2021_1659_MOESM1_ESM.pdf]

# MATERIALS AND METHODS

## Cell culture and transfection

Human MDAMB231$^m$ and murine 4T1 breast cancer cells were transfected with ERO1-Lα CRISPR-Cas9 KO plasmids (SC-401747 for human and SC-424456 for murine, Santa Cruz Biotechnology) with three target-specific guide RNAs (gRNA) of 20 nt. The plasmids were co-transfected with homology-directed repair HDR (SC-401747-HDR for human and SC-424456-HDR for murine cells, Santa Cruz Biotechnology) plasmids, which led to the insertion of puromycin resistance gene and red fluorescent protein (RFP) gene. Cells were seeded 24 h before transfection in a six-well plates and transfected with Fugene HD (E2311, Promega) at a 1:3 DNA/reagent ratio as described in the manufacturer's manual. 72 hours after transfection, cells were detached and seeded in a 10 cm Petri dish and puromycin (2 ug/mL of puromycin for MDAMB231$^m$ and 6 ug/mL for murine 4T1) was added to select positive clones.

## Hypoxic chamber

Human MDAMB231$^m$ and murine 4T1 breast cancer cells were transferred to a hypoxic chamber (Ruskinn Invivo2 400, UK) at 37℃ and maintained in deoxygenated culture medium at the following gas concentrations: $O_2$ 0.1%, $CO_2$ 5% for 48 h.

## Motility assay

HUVEC, MDAMB231$^m$ and 4T1 motility were assessed using modified Boyden chambers and gelatin-coated polycarbonate nucleopore filters (8-µm pore size). Conditioned media from an equal number of WT and ERO1 KO MDAMB231$^m$ cells was used as an attractant to stimulate HUVEC migration. The VEGF antibody, Bevacizumab (250 ng/uL, Roche S.p.A., Milan, Italy) or B20.4.1.1 (B20; 250 ng/ul, Genentech, Inc, South San Francisco, USA) was added to the conditioned medium and incubated throughout the assay. HUVECs were resuspended in DMEM, 0.1% BSA at a concentration of 0, 75×10$^6$/mL, and added to the upper compartment of the chamber. The assay was carried out in 5% CO2 at 37°C for 6h.

To test the migration we used WT and ERO1 KO MDAMB231$^{m}$ and 4T1, the supernatant of NIH3T3 fibroblasts as an attractant and placed in the lower compartment of the chamber. WT and ERO1KO cells were resuspended in high- glucose DMEM with 0.1% BSA at a concentration of $1\times10^{6}$/mL, and added to the upper compartment of the chamber. Assays were carried out in 5% CO2 at 37˚C for 6 hours for MDAMB231$^{m}$ and 4 hours for 4T1.

At the end of the incubation, filters were fixed and stained with Diff-Quik (Marz-Dade, Dundingen, Switzerland) to detect cells adhering to the lower surface, and migrated cells in ten high-power fields for each filter were counted.

**Proteomics sample preparation**

For the secretome analysis, medium without fetal bovine serum was conditioned from ten million MDA-MB231$^{m}$ and collected after 48 hours, then promptly was supplemented with 20 mM NEM and protease inhibitors cocktail (Roche) and concentrated up to 50 uL with Centricon units with a 3-kDa cutoff at 4 °C (Amicon Ultra Centrifugal filters, Millipore, USA) following the manufacturer's protocol. For the analysis of cell pellets, five million MDA-MB231$^{m}$ were resuspended in a buffer containing 8 M urea, 150 mM NaCl, 1.0% Triton, 50 mM Tris, pH 8.0 and protease inhibitor (Roche, Indianapolis, IN). Cell lysates were centrifuged at 12,000 g at 4 °C for 15 min and the supernatants were collected. Equal amounts of proteins (50 μg) for each sample and from three biological replicates were subjected to in-solution digestion.

**In-solution protein digestion**

Proteins were reduced with 5 mM Tris (2-carboxyethyl)phosphine (TCEP) (ThermoFisher Scientific, Waltham, Massachusetts, USA) for 40 minutes at 37 C, then alkylated with 20 mM iodoacetamide (Merck, Burlington, Massachusetts, USA) in the dark and pre-digested with lysyl endopeptidase (Wako Chemicals, Neuss, Germany) at an enzyme to substrate ratio of 1:100 for 4 h at 37C. Digestions were completed by treatment with sequencing-grade porcine trypsin (Promega, Madison, Wisconsin, USA) at an enzyme/substrate ratio of 1:100 for 16 h at 37 C. Peptide mixtures were loaded on a SampliQ C18 ODS cartridge (Agilent Technologies, Santa Clara, California, USA) and eluted

(following manufacturer's instruction). Samples were dried in a vacuum centrifuge, solubilized in 0.1% formic acid, adjusted to 0.5 g/L of peptide concentration (NanoDrop, ThermoFisher Scientific, Waltham, Massachusetts, USA) and immediately analyzed by nano LC-mass spectrometry.

**Mass spectrometry and data analysis**

Peptide samples were analyzed on an Orbitrap Q Exactive mass spectrometer (ThermoFisher Scientific, Waltham, Massachusetts, USA) equipped with a DESI Omni Spray (Prosolia, Waters, Milford, Massachusetts, USA) used in nanospray mode and a nano-flow LC system (Easy-nLC II, ThermoFisher Scientific, Waltham, Massachusetts, USA). Peptides were separated on a picofrit 25 cm x 0.75 µm i.d. column (New Objective, PF360-75-10- N-5, Woburn, Massachusetts, USA) packed in-house with C18 beads. Peptides were eluted with a gradient of 2-60% buffer B (100% ACN, 0.1% formic acid) at a flow rate of 300 nL/min. Label-free proteomics was done in data-dependent acquisition (DDA), MS1 spectra were acquired from 400 to 2000 m/z at a resolution of 70000. The 20 most intense precursors were selected for fragmentation at 30 eV collision energy and the corresponding MS2 spectra were acquired at a resolution of 17500 using singly charged precursor ions and ions of undefinable charged states were excluded from fragmentation.

MaxQuant software (version 1.5.3.30) was used to analyze MS raw files. MS/MS spectra were searched against the human Uniprot FASTA database (Version 2016) and a database of common contaminants (247 entries) by the Andromeda search engine. N-Ethylmaleimide (NEM), Cysteine carbamidomethylation (IAA) and methionine oxidation were applied as variable modification. Enzyme specificity was set to trypsin with a maximum of two missed cleavages and a minimum peptide length of 7 amino acids. A false discovery rate (FDR) of 1% was required for peptides and proteins. Peptide identification was performed with an allowed initial precursor mass deviation of up to 7 ppm and an allowed fragment mass deviation of 20 ppm. Protein identification required at least one razor peptide. A minimum ratio of 1 was required for valid quantification events in MaxQuant's Label-Free Quantification algorithm (MaxLFQ). Data were filtered for common contaminants and peptides only identified by side modification were excluded from further analysis. In addition, a

minimum of two valid quantifications values was required in at least one group of replicates. Bioinformatic analysis was done in the Perseus software environment. The whole mass spectrometry proteomics data have been deposited in the ProteomeXchange Consortium (http://proteomecentral.proteomexchange.org/cgi/GetDataset) via the PRIDE partner repository with the data set identifier PXD019925. NEM and IAA cysteine labeled peptides were considered differently modified in WT and ERO KO cells when detected only in one condition and in two out of three biological replicates/conditions. Proteins detected in secretome samples were searched on the Uniprot database. Statistical differences between experimental groups were analyzed using two-way ANOVA and Mann-Whitney-Wilcoxon Test (JMP pro 13, SAS).

**Protein pathway and network analysis**

Lists of differently secreted IAA labeled proteins and differently expressed proteins were analyzed by pathway analysis using MetaCore analytical suite, version 19.4 (Clarivate Analytics, Philadelphia, Pennsylvania, USA). MetaCore is an integrated analytical suite of algorithms, based on a manually curated database of human protein-protein interactions, transcriptional factors, signaling, metabolism, and bioactive molecules linked to functional processes and diseases. Pathway and network enrichment analyses were used to identify the over-represented protein map and networks against the default background (the entire MetaCore database) ranked by P-value and interpreted in terms of Gene Ontology to deduce top scoring processes regulated by differently expressed proteins.

**MTS**

Six thousand cells/well were plated in 96-well plates. Cells were incubated in MTS [3-(4,5-dimethyltiazol-2-il)-5-(3-carbossymetoxyphenyl)-2-(4-solfophenyl)-2H-tetrazole] and PMS (Phenazine methosulfate) as indicated in the CellTiter 96® Aqueous Non-Radioactive Cell Proliferation Assay (Promega). Acquisitions were done with TECAN infinite M200, using excitation wavelengths at 490 nm.

**Real-time quantitative RT-PCR analysis**

Total RNA was isolated using the RNeasy Mini Kit (Qiagen) following the manufacturer's

instructions. One microgram of total RNA was reverse-transcribed and analyzed using the Applied Biosystems' real-time PCR System and the ΔΔCt method. Relative gene expression in cells was normalized to cyclophilin mRNA levels.

The primer pairs were:

hs-VEGF A_Fw AGG AGG AGG GCA GAA TCA TCA _Rv CTC GAT TGG ATG GCA GTA GCT
hs-VEGF B_Fw GAT CCG GTA CCC GAG CAG TCA G
_Rv CAC CTG CAG GTG TCT GGG TTG A
hs-VEGF C_Fw CAG TGT CAG GCA GCG AAC AA
_Rv CTT CCT GAG CCA GGC ATC TG
hs-VEGFR1_Fw ATC ATT CCG AAG CAA GGT GTG AC
_Rv TCC TTC TAT TAT TGC CAT GCG CT
hs-VEGFR2_Fw TGG GAA CCG GAA CCT CAC TAT C
_Rv GTC TTT TCC TGG GCA CCT TCT ATT
hs-VEGFR3_Fw GTC ACG CTG CGC TCG CAA A
_Rv GTG CAG CAG TGG CGT GGA CA
hs-ERO1_Fw GGC TTC TGG TCA AGG GAC
_Rv TGC TTG CAT GTA GGC CAG ATA
hs-ATF4_Fw GGT TCT CCA GCG ACA AGG
_Rv TCT CCA ACA TCC AAT CTG TCC
hs-CHOP_Fw CAT CAC CAC ACC TGA AAG CA
_Rv TCA GCT GCC ATC TCT GCA
hs-CYCLOPHILIN_Fw GAC CCA ACA CAA ATG GTT CC
_Rv TTT CAC TTT GCC AAA CAC CA

**FLAG-tagged human VEGF$^{121}$ plasmids**

Expression plasmids encoding N-terminally FLAG-tagged human VEGF$^{121}$ (VEGF 1116-1483 of NM_001171628.1) were constructed in the pFLAG-CMV1 vector (Sigma). The FLAG-tagged VEGF$^{121}$ mutants in the cysteines 51, 60 and 116 (C51S, C60S and C116S) were made using Agilent QuikChange II XL Site-Directed Mutagenesis Kit according to manufacturer's instructions.

The mutagenic oligonucleotide primer pairs for each substitution were:

VEGFmut_C51S_Fw GCA TCA GGG GCA CAC TGG ATG GCT TGA AGA T

_Rv ATC TTC AAG CCA TCC AGT GTG CCC CTG ATG C

VEGFmut_C60S_Fw CGT CAT TGC AGC TGC CCC CGC ATC G

_Rv CGA TGC GGG GGC AGC TGC AAT GAC G

VEGFmut_C116S_Fw CCT CGG CTT GTC ACT TTT TTC TTG TCT TGC TCT ATC TTT CT

_Rv AGA AAG ATA GAG CAA GAC AAG AAA AAA GTG ACA AGC CGA GG.

All resulting constructs were verified by Sanger sequencing.

**Western blotting**

Cells were lysed in cold buffer containing 150 mM NaCl, 20 mM HEPES pH 7.5, 10 mM EDTA and 1% Triton X100, supplemented with a protease inhibitors cocktail (Roche) and 20 mM NEM. The protein concentration was determined by a standard BCA assay (Pierce). Samples with equal protein concentration were mixed with non-reducing Laemmli buffer (62.5 mM Tris-HCl pH 6.8, 2% SDS, 10% glycerol and 0.01% bromophenol blue) and heated for 5 min at 95°C. For reducing SDS-PAGE, samples were supplemented with 100 mM DTT. Protein samples separated by either reducing or non-reducing SDS-PAGE were then transferred to Protran nitrocellulose membrane (Merck) and probed with the following antibodies: monoclonal mouse anti-Actin (MAB1501, Sigma Aldrich), monoclonal mouse anti-KDEL (ADI-SPA-827, Enzo life Sciences), monoclonal mouse anti-FLAG M2 (F3165, Sigma Aldrich), and polyclonal rabbit anti-ERO1 alpha [9].

**Immunoprecipitation**

WT and ERO1 KO HeLa cells were lysed in cold lysis buffer supplemented with a protease inhibitors cocktail (Roche), 10 mM calcium and 20 mM NEM. Conditioned media were treated with 20 NEM and protease inhibitors cocktail before being concentrated with Amicon Ultra-15 (3 kDa). 10 mM calcium was added to the concentrated media. Samples containing 1-2 mg of total protein were pre-cleared using SureBeads protein G magnetic beads (Bio-Rad Laboratories) for 1 h and incubated overnight with 20-30 uL of FLAG-M1 (Sigma-Aldrich). The FLAG-tagged VEGF was finally eluted in Lemmli buffer without any DTT.

**Surface Plasmon Resonance (SPR)**

The ProteOn XPR36 Protein Interaction Array system (Bio-Rad Laboratories, Hercules, CA) was used for these studies. VEGF neutralizing antibodies B20 and Bevacizumab as well as IgG from

human serum (I4506, Sigma) were covalently immobilized in three parallel channels of the same sensor chip (GLC, Bio-Rad) by amine coupling chemistry [2].

Briefly, after pre-activation of the chip surfaces, the three antibodies were flowed for 5 min at 30 µg/mL in acetate buffer pH 5, followed by surface inactivation with 1 M ethanolamine. The levels of immobilization of antibodies were similar, around 6000 Resonance Units (RU, where 1000 RU = 1 ng/mm2).

For the binding studies, the ProteOn XPR36 fluidic system was rotated 90° so that CM from different cells (FLAG-VEGF$^{121}$-WT, -ERO1KO HeLa or WT, ERO1KO MDAMB231$^{m}$ diluted two-fold) were injected simultaneously on immobilized B20, bevacizumab, and IgG (as a control for non-specific binding). CMs were flowed for 5-10 min (as indicated), at a rate of 30 µL/min. The running buffer was composed of 10 mM phosphate buffer with 150 mM NaCl and 0.005% Tween 20 (PBST, pH 7.4).

The non-specific SPR signal measured on the reference surface (immobilized IgG), due to supernatant components, was subtracted from the SPR signal measured on immobilized B20 or bevacizumab to determine the specific binding due to FLAG-VEGF$^{121}$ or endogenous VEGF.

The signal related to FLAG-VEGF$^{121}$ was further confirmed by injecting anti-FLAG antibody or Bevacizumab (10 µg/mL), soon after the injection of cell supernatants [2].

All SPR assays were run at a rate of 30 µL/min at 25°C. The sensorgrams (time course of the SPR signal in RU) were normalized to a baseline of 0.

**Breast tumor models**

Medullary breast adenocarcinoma cells, E0771, were inoculated orthotopically (1x10$^{6}$) in the mammary fat pad (m. f. p.) of WT and ERO1 KO mice.

Briefly, in anesthetized mice, the skin was disinfected with Betadine and a small incision was made in the skin adjacent to the fourth mammary fat pad was created. Tumor cell suspension (10 µL) was injected into the fourth mammary gland fat pad using a Hamilton syringe with a 26-gauge needle; after cell transplant, the wound was closed with metal wound clips. When tumors were palpable, the

tumor volume was measured with Vernier caliper once a week and calculated according to the formula *D* × *d2/2*, where *D* is the largest diameter of the tumor and *d* the smallest one. The mice whose tumors did not reach a palpable size were excluded by the analysis. When the primary tumor reached 10% of body weight, the mice were euthanized. Primary tumors and tissues were collected and metastases quantified by bioluminescence imaging (BLI). Briefly, D-luciferin (150 mg/kg, i.p, Caliper Lifescience) injected mice were scanned after 10 minutes with IVIS Lumina Series III XRMS (Perkin Elmer). Images were analyzed with Living Image software (Perkin Elmer) and tumor and metastasis burden were expressed as total flux (photons/sec).

G*Power, version 3.1.9.2, was used to calculate the power analysis. For spontaneous metastasis studies, WT and ERO1 KO 4T1 cell suspensions were inoculated orthotopically ($1x10^6$) in the m. f. p. as described above.

When the primary tumors reached around 150-200 $mm^3$ ($1mm^3$=1mg), tumors were surgically resected and stored snap-frozen or formalin-fixed paraffin-embedded (FFPE) for further analysis. The presence and progression of metastases were followed by BLI once a week.

All animals were euthanized 19 days after primary tumor removal to compare the extent of lymph node and lung metastases between groups. *Ex vivo* BLI of the lung was used to quantify metastatic spread. Lymph nodes and lungs were collected in FFPE for further analysis.

Artificial metastases were obtained by injecting WT and ERO1KO 4T1 ($1x10^6$) into the caudal tail vein (i.v.) of BALB/cByJ mice. *In vivo* BLI was used to follow metastatic progression, as described above. All animals were euthanized 21 days after transplant to compare the extent of lymph nodes and lung metastases between groups. *Ex vivo* BLI of the lung was used to quantify metastatic spread.

WT- and ERO1KO-MDAMB231$^m$ cell suspensions were inoculated orthotopically ($2x10^6$) in the m.f.p. of SCID mice as described above.

When the primary tumor reached around 200-250 $mm^3$, it was surgically resected and stored snap frozen and FFPE. The presence and progression of metastases were followed by BLI. Twenty-one

days after surgery mice were sacrificed and the extent of metastases to lymph nodes and lungs quantified by BLI.

**Histology and immunohistochemistry**

Subcutaneous tumors from MDAMB231$^{m}$ were collected and fixed in 10% neutral buffered formalin and paraffin-embedded for histological analyses. 4 µm serial sections from each sample were routinely stained with Hematoxylin and Eosin (H&E) and examined by immunohistochemistry to detect tumoral vessels, with the rat anti-CD31 antibody (clone SZ31, Dianova, DIA310, dilution 1:100).

For immunohistochemistry, deparaffinization, rehydration and antigen retrieval were done in a single step method: sections were immersed for 40 min at 94°C in a pH 9 buffer solution (Dewax and HIER Buffer H, Thermo Scientific). Endogenous peroxidase activity was blocked by incubating sections in 3% $H_2O_2$ for 15 min. To reduce nonspecific background staining, slides were rinsed and treated for 30 min with 10% rabbit normal serum and then incubated with the primary antibody for 1 hour at room temperature.

A biotinylated secondary antibody (Vector Laboratories) was then added for 30 min and sections were labeled by the avidin-biotin-peroxidase (ABC) procedure with a commercial immunoperoxidase kit (Vectastain Standard Elite, Vector Laboratories). The immunoreaction was revealed with 3,3'-diaminobenzidine substrate (DAB, Vector Laboratories) for 5 min and sections were counterstained with Meyer's hematoxylin.

Mitotic count was obtained by counting the number of mitoses in 3 random 400x microscopic fields on H&E slides. The tumor-related vasculature was evaluated by counting the number of CD31-positive vessels (Microvessel Density, MVD) and by calculating the percentage of CD31-immunopositive area (Endothelial Area, EA) in 3 200x microscopic fields, randomly selected throughout the neoplastic tissue, using ImageJ software.

**Trascriptomics**

RNAseq Cancer Cell Line Encyclopedia [6] ERO1 mRNA z-score expression levels of breast cancer cell lines were retrieved from the CBioPortal for Cancer Genomics website (https://www.cbioportal.org/) [4]. Cell lines were classified as Basal or Luminal as reported in [1]. Raw counts for BRCA TCGA samples were retrieved using the TCGA biolinks package [3] in the R/Bioconductor environment [5]. Raw read counts were normalized using a Variance Stabilizing Transformation (VST), implemented in the R/Bioconductor DESeq2 package [7]. Expression data were then reported as $\log_2$CPM. Statistical analysis of the differences of the single genes expression levels in cancer subgroups was performed using one-way ANOVA followed by Tukey's test. Coexpression among the genes was measured using the Pearson correlation coefficient and t-statistics. Correlation heatmaps were generated using the ClustVis web-tool [8].

**Survival analyses**

Clinical data for the analysis of disease-free interval and overall survival or time to metastasis of breast cancer patients from TCGA and the Metastatic Breast Cancer project (Provisional, February 2020), respectively, along with ERO1 z-score expression were downloaded from the CBioPortal. Cox proportional hazard analysis was performed using the Survival package in R/Bioconductor.

**References:**

1 Bolis M, Garattini E, Paroni G, Zanetti A, Kurosaki M, Castrignano T *et al*. Network-guided modeling allows tumor-type independent prediction of sensitivity to all-trans-retinoic acid. Ann Oncol 2017; 28: 611-621.

2 Canovi M, Lucchetti J, Stravalaci M, Valentino S, Bottazzi B, Salmona M *et al*. A new surface plasmon resonance-based immunoassay for rapid, reproducible and sensitive quantification of pentraxin-3 in human plasma. Sensors (Basel) 2014; 14: 10864-10875.

3 Colaprico A, Silva TC, Olsen C, Garofano L, Cava C, Garolini D *et al*. TCGAbiolinks: an R/Bioconductor package for integrative analysis of TCGA data. Nucleic Acids Res 2016; 44: e71.

4 Gao J, Aksoy BA, Dogrusoz U, Dresdner G, Gross B, Sumer SO *et al*. Integrative analysis of complex cancer genomics and clinical profiles using the cBioPortal. Sci Signal 2013; 6: pl1.

5 Gentleman RC, Carey VJ, Bates DM, Bolstad B, Dettling M, Dudoit S *et al*. Bioconductor: open software development for computational biology and bioinformatics. Genome Biol 2004; 5: R80.

6 Ghandi M, Huang FW, Jane-Valbuena J, Kryukov GV, Lo CC, McDonald ER, 3rd *et al*. Next-generation characterization of the Cancer Cell Line Encyclopedia. Nature 2019; 569: 503-508.

7 Love MI, Huber W, Anders S. Moderated estimation of fold change and dispersion for RNA-seq data with DESeq2. Genome Biol 2014; 15: 550.

8 Metsalu T, Vilo J. ClustVis: a web tool for visualizing clustering of multivariate data using Principal Component Analysis and heatmap. Nucleic Acids Res 2015; 43: W566-570.

9 Zito E, Chin KT, Blais J, Harding HP, Ron D. ERO1-beta, a pancreas-specific disulfide oxidase, promotes insulin biogenesis and glucose homeostasis. J Cell Biol 2010; 188: 821-832.

**Figure legends:**

**Supplementary Figure 1**

A) Bar graph of ERO1 expression in different luminal and basal cancer cell lines. Below, box plot of ERO1 expression across 22 basal and 19 luminal cell lines. ERO1 levels are obtained through the analysis of RNAseq Cancer Cell Line Encyclopedia datasets (PAM50 mean_ERO1, sd_ERO11: Basal 0.3075709± 0.5492449, Luminal -0.2819400 ± 0.2329616 p value t-test 7.045e-05). B) Immunoblot of ERO1 in normoxic and hypoxic conditions ($O_2$ levels at 0.1%) across luminal and basal breast cancer cells. Actin is used as a loading control.

**Supplementary Figure 2**

A) MDAMB231$^{m}$ cell line was obtained after serial passages in the mammary fat pad of cells collected from the metastatic lymph nodes of MDA-MB231. MDAMB231$^{m}$ had a more rapid tumor growth (as indicated by the growth curve) and a higher primary tumor take (as indicated by the table, below) compared to the parental MDAMB231cell line. After surgery of the primary tumor, the metastases to axillary lymph nodes and lungs occurred faster in MDAMB231$^{m}$ than MDAMB231, as indicated by the table.

B) Bar graphs of real-time RT-PCR analysis of mRNAs from WT and ERO1 KO MDAMB231$^{m}$ cells undergoing a time course of hypoxia as indicated (N=3).

C) Non-reducing and reducing FLAG Immunoblot of FLAG-tagged VEGF$^{121}$ (WT) immunopurified with FLAG-M1 antibody from conditioned media of WT and ERO1 KO MDAMB231$^{m}$ cells that were mock-transfected or transfected with expression plasmids containing FLAG-tagged VEGFA$^{121}$. On the right, the dot plot indicates the ratio of dimer to monomer of VEGFA$^{121}$ in WT and ERO1 KO cells in two different experiments. The ratio of ERO1 KO was set to 1.

**Supplementary Figure 3**

A) ERO1 immunoblot of 4T1 clones which were screened for CRISPR/CAS9-mediated editing events (clones 1, 2, 4, 5, 6, 7, 8, 9 are ERO1 KO). Actin is used as a loading control. B) Dot plots

indicating the number of WT and ERO1 KO cells migrated from 10 different fields of three different Boyden chambers. C) Growth curve (MTS) of an equal number of WT and ERO1 KO 4T1 cells (N=4). D) Bioluminescence signals of primary breast tumors and E) lungs from representative WT and ERO1 KO 4T1-orthotopically injected mice. Below, bar graphs on logarithmic scale of the bioluminescence counts of the primary tumor and lungs (N=4). F) Bioluminescence signals of artificial metastases from representative mice tail vein-injected with WT and ERO1 KO 4T1. On the left, dot plots of the bioluminescence signal from artificial metastases (N=8-9). G) Growth curve of primary breast tumors and H) lung metastases of E0771-orthotopically injected in WT and ERO1 KO mice (N=6-7).

**Supplementary Figure 4**

A) Box plots of the expression of the indicated genes in basal, luminal, Her2+, breast cancer and normal breast samples from TCGA dataset (RNAseq).

B) Pairwise comparisons of each of the indicated gene products among the different breast cancer subtypes. Values indicate the significance of the comparison (Tukey's test, performed after one-way ANOVA). Background colors indicate the direction of the differences (orange: higher, blue: lower). Full colors: significance ($p<0.05$), light colors: trend ($0.10<p<=0.05$). White background: no significant difference.

**Supplementary Table 1**

Process network analysis report (MetaCore analytical suite version 19.4) obtained by statistically significant altered intracellular proteins (nLC-MS/MS proteomic analysis, Wilcoxon Mann Whitney test, $p<0.05$) between ERO1 KO vs WT MDA-MB231$^{m}$ in hypoxic conditions.

**Supplementary Table 2**

Enrichment analysis report (MetaCore analytical suite version 19.4) using all identified secreted proteins (nLC-MS/MS proteomic analysis) in ERO1 KO and WT MDA-MB231$^{m}$ in hypoxic conditions. HIF-1 secreted proteins are highlighted in bold.

Sup.1

A

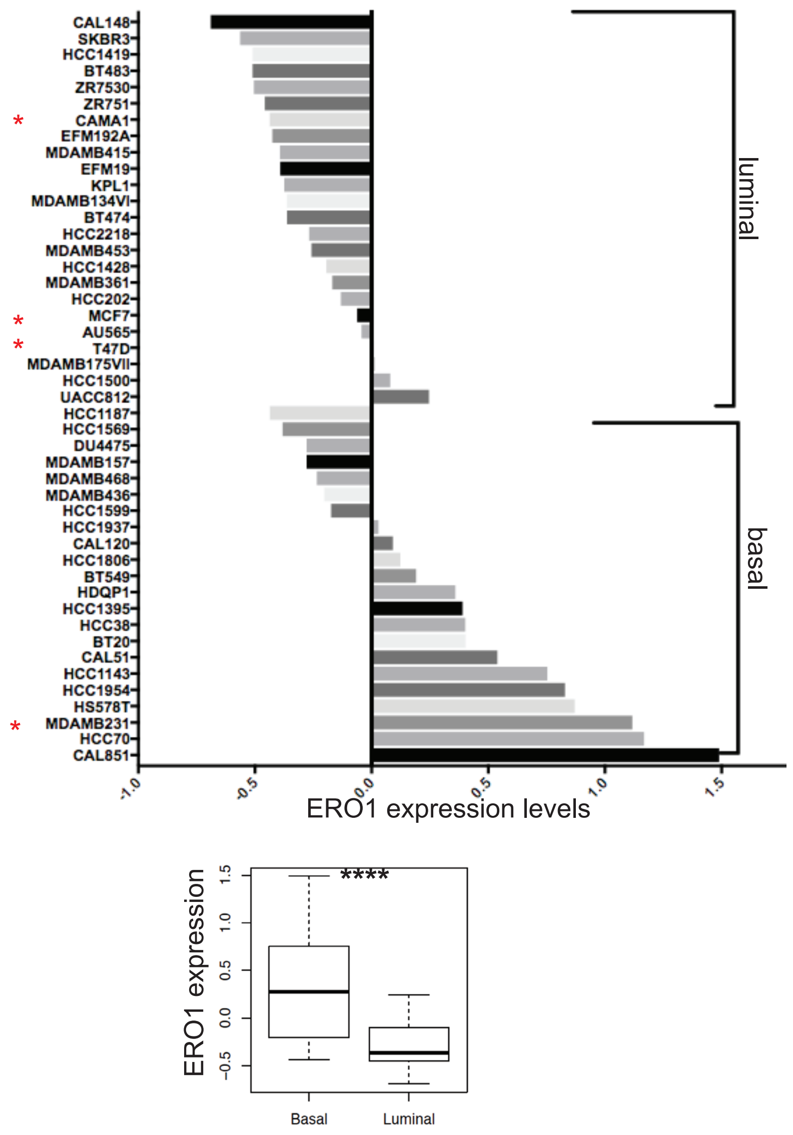
CAL148
SKBR3
HCC1419
BT483
ZR7530
ZR751
CAMA1
EFM192A
MDAMB415
EFM19
KPL1
MDAMB134VI
BT474
HCC2218
MDAMB453
HCC1428
MDAMB361
HCC202
MCF7
AU565
T47D
MDAMB175VII
HCC1500
UACC812
HCC1187
HCC1569
DU4475
MDAMB157
MDAMB468
MDAMB436
HCC1599
HCC1937
CAL120
HCC1806
BT549
HDQP1
HCC1395
HCC38
BT20
CAL51
HCC1143
HCC1954
HS578T
MDAMB231
HCC70
CAL851
luminal
basal
-1.0
-0.5
0.0
0.5
1.0
1.5
ERO1 expression levels
****
ERO1 expression
-0.5
0.0
0.5
1.0
1.5
Basal
Luminal

B

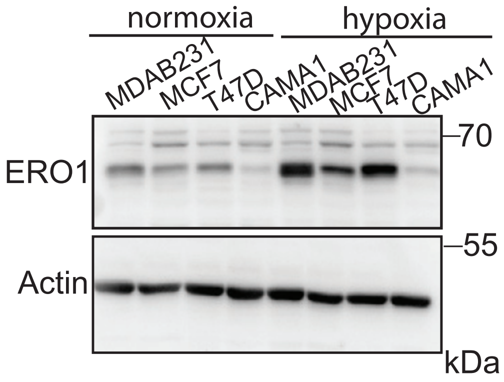
normoxia
hypoxia
MDAB231
MCF7
T47D
CAMA1
MDAB231
MCF7
T47D
CAMA1
ERO1
Actin
70
55
kDa

**A**

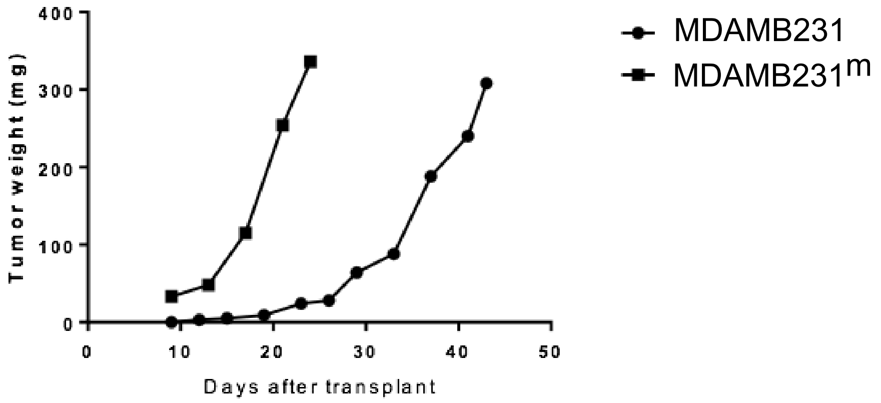


| | MDAMB231 | MDAMB231m |
|---|---|---|
| Surgery for primary tumor removal (days) | 43 | 24 |
| Primary tumors | 4/5 | 5/5 |
| Lymph node metastasis (days post surgery) | 4-6 weeks | 18 days |
| Lung metastsis | 4/4 | 5/5 |

**B**

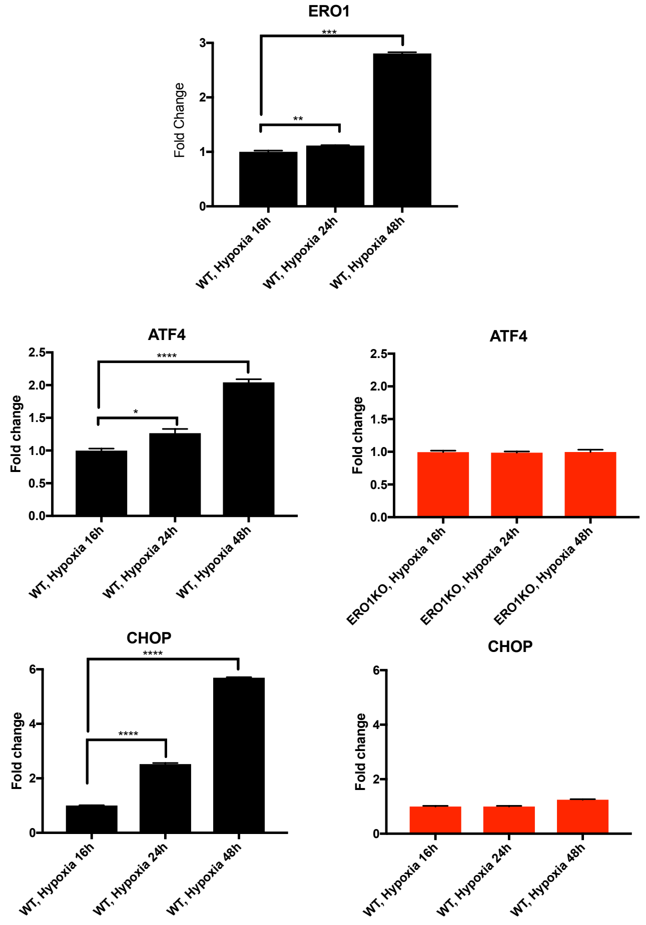


**C**

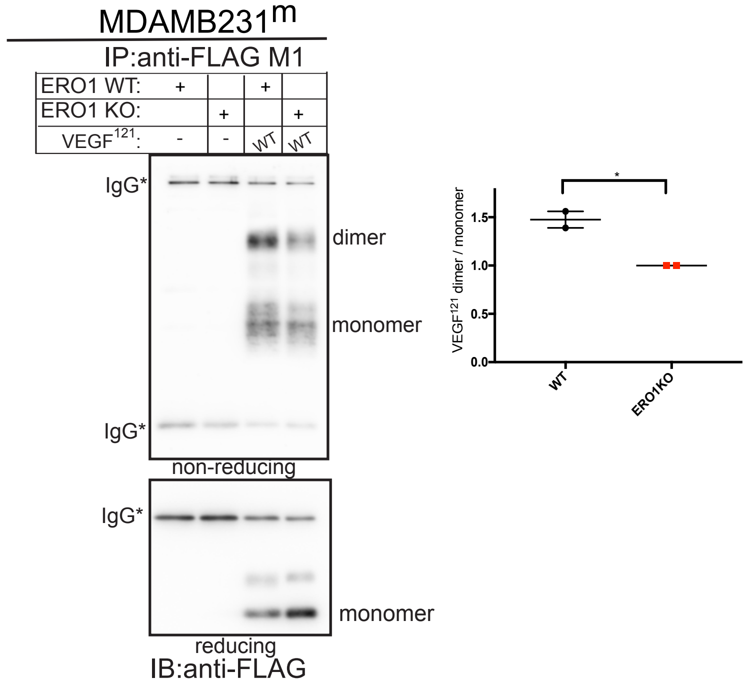

Sup. 3

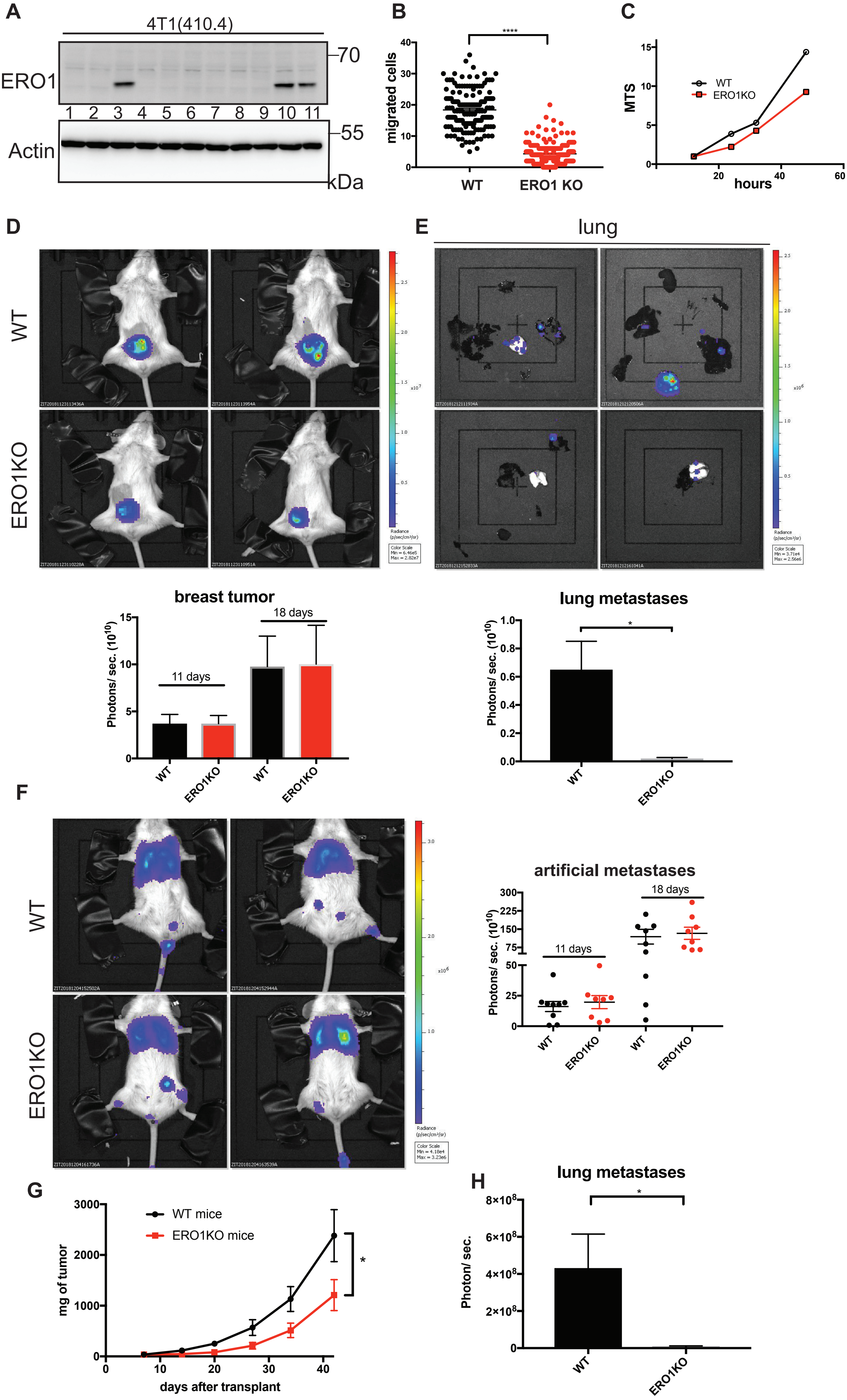

A
4T1(410.4)
ERO1
Actin
1 2 3 4 5 6 7 8 9 10 11
70
55
kDa
B
migrated cells
****
WT
ERO1 KO
C
MTS
WT
ERO1KO
hours
D
WT
ERO1KO
E
lung
breast tumor
Photons/ sec. (10^10)
11 days
18 days
WT
ERO1KO
lung metastases
*
F
artificial metastases
G
WT mice
ERO1KO mice
mg of tumor
days after transplant
H
Photon/ sec.

A

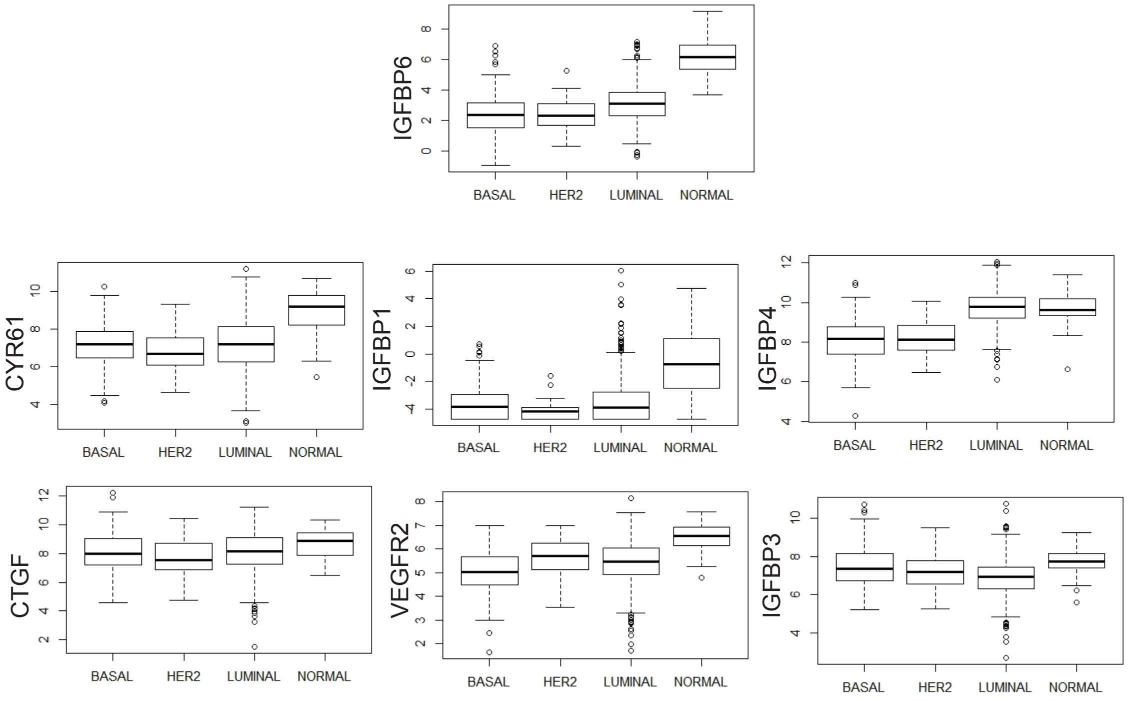


B

| GENE | HER2 vs BASAL | LUMINAL vs BASAL | NORMAL vs BASAL | LUMINAL vs HER2 | NORMAL vs HER2 | NORMAL vs LUMINAL |
|---|---|---|---|---|---|---|
| ERO1A | 5,0E-07 | <1E-07 | <1E-07 | <1E-07 | <1E-07 | 1,6E-06 |
| VEGFA | 9,8E-01 | <1E-07 | <1E-07 | <1E-07 | <1E-07 | 2,4E-03 |
| SERPINE1 | 3,0E-01 | 2,5E-03 | 5,5E-05 | 1,0E+00 | 5,6E-06 | <1E-07 |
| EIF2S1 | 5,3E-02 | 2,6E-01 | 3,6E-02 | 5,6E-04 | 8,9E-05 | 3,0E-01 |
| ATF4 | 5,5E-02 | <1E-07 | 3,7E-04 | 1,9E-04 | 8,9E-01 | 1,5E-04 |
| CHOP (DDIT3) | 2,5E-01 | 1,8E-03 | 5,4E-02 | 1,0E+00 | 1,0E+00 | 1,0E+00 |
| PERK (EIF2AK3) | 1,0E-07 | 2,7E-05 | 3,0E-01 | 1,7E-03 | <1E-07 | 2,0E-07 |
| MMP1 | 4,5E-01 | <1E-07 | <1E-07 | <1E-07 | <1E-07 | <1E-07 |
| PRDX4 | 1,2E-04 | <1E-07 | <1E-07 | <1E-07 | <1E-07 | <1E-07 |
| LGALS1 | 1,5E-01 | 5,1E-04 | 9,3E-01 | 1,0E+00 | 8,2E-02 | 1,1E-03 |
| TFRC | 3,7E-01 | <1E-07 | <1E-07 | <1E-07 | <1E-07 | 1,0E-07 |
| PLAU | 5,5E-02 | 6,7E-02 | <1E-07 | 1,2E-04 | 0,0E+00 | <1E-07 |
| TGFB1 | 7,9E-01 | <1E-07 | 1,9E-04 | 6,0E-02 | 4,2E-04 | <1E-07 |
