# Supplementary material for: The ER stress response mediator ERO1 triggers cancer metastasis by favoring the angiogenic switch in hypoxic conditions
Source: Oncogene. 2021 Feb 2;40(9):1721–36. doi: 10.1038/s41388-021-01659-y (PMC7932925; doi:10.1038/s41388-021-01659-y)
Supplement: Supplementary file 2 — Supplemental table 1 [file 41388_2021_1659_MOESM2_ESM.pdf]

**Process network analysis report significantly altered proteins in ERO1 KO vs WT hypoxic condition (Wilcoxon Mann Whitney test, p<0.05).**

| Enrichment by Process Networks | | | | | All_KO vs WT I | | | |
|---|---|---|---|---|---|---|---|---|
| # | Networks | Total | pValue | Min FDR | p-value | FDR | In Data | Network Objects from Active Data |
| 1 | Protein folding_Response to unfolded proteins | 71 | 1,962E-09 | 2,080E-07 | 1,962E-09 | 2,080E-07 | 11 | Endoplasmin, HSP90 alpha, HSP90 beta, HSC70, GRP78, DNAJC3, HSP60, HYOU1, HSPA2, VCP, Hdj-2 |
| 2 | Protein folding_Folding in normal condition | 119 | 4,751E-07 | 2,518E-05 | 4,751E-07 | 2,518E-05 | 11 | Endoplasmin, HSP90 alpha, HSP90 beta, Sti1, HSC70, DNAJC3, HSP60, HYOU1, HSPA2, TCP1-beta, Hdj-2 |
| 3 | Protein folding_ER and cytoplasm | 44 | 2,395E-05 | 8,463E-04 | 2,395E-05 | 8,463E-04 | 6 | UGCGL1, HSC70, GRP78, DNAJC3, HYOU1, VCP |
| 4 | Translation_Elongation-Termination | 233 | 6,212E-05 | 1,646E-03 | 6,212E-05 | 1,646E-03 | 12 | DDX1, RPL7A, eEF1D, SRP72, IleRS, GlyRS, DARS, TyrRS, ValRS2, RP40, RPL14, MARS |
| 5 | Cytoskeleton_Intermediate filaments | 81 | 1,001E-04 | 2,122E-03 | 1,001E-04 | 2,122E-03 | 7 | TMPOA, Plectin 1, Vimentin, Keratin 18, Lamin B1, TMPOB, Lamin A/C |
| 6 | Immune response_Phagosome in antigen presentation | 243 | 3,957E-04 | 6,990E-03 | 3,957E-04 | 6,990E-03 | 11 | Endoplasmin, PSMD14, HSP90 alpha, HSP90 beta, Profilin I, Gelsolin, CDC42, GRP78, NF-kB p65/p65, PSMD11, VCP |
| 7 | Transcription_mRNA processing | 160 | 1,353E-03 | 2,049E-02 | 1,353E-03 | 2,049E-02 | 8 | hnRNP A2, Prp8, DDX1, SNRP116, SF3A1, DDX39, Mago nashi, SF3B1 |
| 8 | Immune response_Antigen presentation | 197 | 4,925E-03 | 6,526E-02 | 4,925E-03 | 6,526E-02 | 8 | Endoplasmin, PSMD14, HSP90 alpha, HSP90 beta, ECM29, GRP78, NF-kB p65/p65, PSMD11 |
| 9 | Protein folding_Protein folding nucleus | 58 | 7,549E-03 | 7,790E-02 | 7,549E-03 | 7,790E-02 | 4 | HSC70, GRP78, HYOU1, DJ-1 |
| 10 | Cell adhesion_Integrin-mediated cell-matrix adhesion | 214 | 8,021E-03 | 7,790E-02 | 8,021E-03 | 7,790E-02 | 8 | TGM2, Filamin C, Profilin I, ITGA6, ITGB4, ITGA2, CDC42, Cyr61 |
