# Supplementary material for: The ER stress response mediator ERO1 triggers cancer metastasis by favoring the angiogenic switch in hypoxic conditions
Source: Oncogene. 2021 Feb 2;40(9):1721–36. doi: 10.1038/s41388-021-01659-y (PMC7932925; doi:10.1038/s41388-021-01659-y)
Supplement: Supplementary file 3 — Supplemental table 2 [file 41388_2021_1659_MOESM3_ESM.pdf]

Enrichment analysis report using all identified secreted proteins in ERO1 KO and WT in hypoxic condition

Enrichment by Pathway Maps					Secteted all Hx_genelist			
**#**	**Maps**	**Total**	**pValue**	**Min FDR**	**p-value**	**FDR**	**In Data**	**Network Objects from Active Data**
1	Beta-catenin-dependent transcription regulation in colorectal cancer	36	7,989E-13	7,022E-10	7,989E-13	7,022E-10	13	CD44, ELAVL1 (HuR), Tenascin-C, CD44 soluble, PLAU (UPA), LAMC2, LAMC2 (80kDa), LAMC2 (100kDa), L1CAM, Calcyclin, MMP-14, CD44 (EXT), Lamin A/C
2	CFTR folding and maturation (normal and CF)	24	6,953E-11	2,063E-08	6,953E-11	2,063E-08	10	Csp, ERp29, Sti1, HSP40, HSP105, HSP90 alpha, BAG-3, HSP90 beta, PARP-1, GANAB
3	Transcription_HIF-1 targets	95	7,042E-11	2,063E-08	7,042E-11	2,063E-08	17	G3P2, **TfR1 (TFRC)**, LDHA, **IBP1**, Nucleophosmin, **Galectin-1 (LGALS1)**, Thrombospondin 1, ALDOC, **PLAU**, **PAI1 (SERPINE1)** PGK1, ENO1,**TGFB1**, ALDOA, **CTGF**, PKM2, Lysyl oxidase, **IBP3**
4	Cell adhesion_ECM remodeling	55	3,353E-10	7,368E-08	3,353E-10	7,368E-08	13	IBP4, CD44, TIMP2, PLAT (TPA), MSN (moesin), Fibronectin, MMP-1, PAI1, MMP-13, PLAU (UPA), TIMP1, MMP-14, VIL2 (ezrin)
5	Role of metalloproteases and heparanase in progression of pancreatic cancer	33	4,847E-08	8,520E-06	4,847E-08	8,520E-06	9	CD44, Alpha 1-antitrypsin, TIMP2, MMP-1, VEGF-A, CD44 soluble, TIMP1, MMP-14, CD44 (EXT)
6	Role of TGF-beta 1 in fibrosis development after myocardial infarction	38	1,846E-07	2,496E-05	1,846E-07	2,496E-05	9	COL1A1, TIMP2, Thrombospondin 1, TGFB1, Fibronectin, Tenascin-C, MMP-1, PAI1, TIMP1, CTGF
7	Gamma-secretase proteolytic targets	76	1,988E-07	2,496E-05	1,988E-07	2,496E-05	12	CD44, alphaAPPs, APP-C99, APP-C83 (CTF), APP-C59 (AICD), CD44 soluble, Amyloid beta 40, CD44 (ICD), ADAM9, CD44 (EXT), Amyloid beta 42, APP
8	Glycolysis and gluconeogenesis	94	2,093E-06	2,300E-04	2,093E-06	2,300E-04	12	G3P2, LDHA, TPI1, ALDOC, MDH1, PGK1, ENO1, ALDOA, PGAM1, LDHB, PKM2, PGAM4
9	FAK1 signaling in melanoma	42	5,266E-06	5,143E-04	5,266E-06	5,143E-04	8	TIMP2, Fibronectin, VEGF-A, CRK, RelA (p65 NF-kB subunit), PLAU (UPA), Syntenin 1, MMP-14
10	Cell cycle_Role of 14-3-3 proteins in cell cycle regulation	22	9,082E-06	7,983E-04	9,082E-06	7,983E-04	6	14-3-3 gamma, 14-3-3 beta/alpha, 14-3-3 theta, 14-3-3 epsilon, 14-3-3 sigma, 14-3-3 zeta/delta
	HIF-1 target secreted proteins are highlighted in bold							
